# Machine learning-based prediction of distant metastasis risk in invasive ductal carcinoma of the breast

**DOI:** 10.1371/journal.pone.0310410

**Published:** 2025-02-26

**Authors:** Jingru Dong, Ruijiao Lei, Feiyang Ma, Lu Yu, Lanlan Wang, Shangzhi Xu, Yunhua Hu, Jialin Sun, Wenwen Zhang, Haixia Wang, Li Zhang

**Affiliations:** Shihezi University Medical College School of Medical College, Shihezi University, Shihezi, Xinjiang, China; Shahid Beheshti University of Medical Sciences, ISLAMIC REPUBLIC OF IRAN

## Abstract

More than 90% of deaths due to breast cancer (BC) are due to metastasis-related complications, with invasive ductal carcinoma (IDC) of the breast being the most common pathologic type of breast cancer and highly susceptible to metastasis to distant organs. BC patients who develop cancer metastases are more likely to have a poor prognosis and poor quality of life, so it is extremely important to recognize and diagnose whether distant metastases have occurred in IDC as early as possible. In this study, we develop a non-invasive breast cancer classification system for detecting cancer metastasis. We used Anaconda-Jupyter notebooks to develop various Python programming modules for text mining, data processing, and machine learning (ML) methods. A risk prediction model was constructed based on four algorithms: Random Forest, XGBoost, Logistic Regression, and SVM. Additionally, we developed a hybrid model based on a voting mechanism using these four algorithms as the base models. The models were compared and evaluated by the following metrics: accuracy, precision, recall, F1-score, and area under the ROC curve (AUC) values. The experimental results show that the hybrid model based on the voting mechanism exhibits the best prediction performance (accuracy: 0.867, precision: 0.929, recall: 0.805, F1-score: 0.856, AUC: 0.94). This stable risk prediction model provides a valuable reference support for doctors in assessing and diagnosing the risk of IDC hematogenous metastasis. It also improves the work efficiency of doctors and strives to provide patients with increased chances of survival.

## 1 Introduction

Breast cancer (BC) is the most common malignant tumor and the main cause of cancer-related deaths in women worldwide [1]. The World Health Organization (WHO) International Agency for Research on Cancer (IARC) released the global cancer burden data in 2020 [2], and the latest statistical report data show that the number of new cases of BC worldwide has exceeded 2.26 million, exceeding the number of cases of lung cancer for the first time. BC has thus been crowned the world’s No. 1 cancer, replacing lung cancer, which had been top of the list for many years [2]. In recent years, the incidence and mortality rates of female BC have been increasing yearly, and the proportion of incidence and mortality of all malignant tumors in women has also increased [3], which seriously affects women’s physical and mental health. It is projected that by the year 2040, the number of breast cancer patients will increase to 4.07 million, with 1.4 million fatalities [4].

Despite the good therapeutic results and prognosis achieved in the treatment of early-stage BC, the prognosis of patients with metastatic BC is still poor. More than 90% of deaths in patients with BC are not caused by the primary tumor but by distant metastasis of the primary tumor [5, 6]. Infiltrating Ductal Carcinoma (IDC) is the most common subtype among BC, accounting for about 80% of all BC cases [7]. IDC is also the most common form of invasive BC. This pathologic tissue type of BC has a poor prognosis and is highly susceptible to axillary lymph node and distant organ metastases, and the five-year survival rate of patients with distant metastasis from invasive ductal carcinoma is only 27.4%. Therefore, early detection of distant metastasis in BC can help develop individualized treatment plans, which has specific significance for predicting patient survival time and improving patient prognosis [8]. Cancer metastasis risk assessment is important to reduce overall BC mortality. Appropriate risk assessment is a major factor in BC management that determines patient survivability [9].

The successful introduction of information and communication technologies (ICT) in medical practice is an important stake in the renovation of the health system and more precisely in cancer care [10]. With the development of artificial intelligence, machine learning has shown a very strong data mining ability and has been applied to all walks of life. Machine learning is a field that focuses on the learning aspect of artificial intelligence by developing algorithms that best represent a set of data to guide computers to learn from the data and then make the best decisions and predictions based on the results of data analysis [11]. Combining machine learning with medical problems and integrating this development, more intelligent auxiliary diagnosis methods can be proposed to increase treatment time and cure opportunities for patients. In recent years, machine learning has proven to be of great value in BC diagnosis and survival, prognosis prediction, and other aspects [12, 13]. The use of machine learning algorithms to predict the survival risk of BC is currently a research hotspot at the intersection of artificial intelligence and medicine [14].

Although some BC prediction models based on the SEER database have been developed previously (Table 1), few studies have attempted to use the ML algorithm to predict the risk of distant IDC transfers. In this study, machine learning algorithms was used instead of traditional statistical analysis methods to process the clinical characteristics data of patients with IDC, and a machine learning-based predictive model was developed to discover the relationship between certain independent variables and the likelihood of patients with IDC developing distant organ metastasis. Focusing on the predictive models of four algorithms: Logistic Regression, SVM, Random Forest and XGBoost, and using them as base models for integration to construct hybrid models. A ten-fold cross-validation was used to compare the accuracy, recall, precision, and AUC value of each model to predict the risk of cancer metastasis in patients with IDC, thereby assisting clinicians in making more rational clinical decisions and enabling patients to receive treatment earlier, which is useful for breast surgeons to quickly and accurately determine whether cancer metastasis has occurred in patients with IDC.

**Table 1:** Comparison of information on predicting survival outcomes or metastasis of cancer patients using machine learning algorithms in the past.

Authors	Prediction model	Objective	Result
Ahmad et al., 2022 [15]	NB, SVM, MLP, J 48 and RF.	A 10-fold cross validation was used.	NB gave the best accuracy. 97.3%.
Islam et al., 2020 [16]	SVM, KNN, RF, ANN, and LR	A 10-fold cross validation was employed.	ANN yielded the highest accuracy. 98.57%.
Choudhury et al., 2021 [17]	MLP, VP, CC, KLR, SGD, AdaBoost, VFDT, SVM	To diagnose and predict the cancer prognosis of Malignant Pleural Mesothelioma as early as possible (MPM).	AdaBoost have the highest accuracy. 71.29%.
Alfian et al., 2022 [18]	SVM	Predicting Breast Cancer from Risk Factors Using SVM and Extra-TreesBased Feature Selection Method.	80.23%
Afolayan et al., 2022 [19]	DT	Breast cancer detection using particle swarm optimization and decision tree machine learning technique.	92.26%
Seo et al., 2022 [20]	SVM	Scaling multi-instance support vector machine to breast cancer detection on the BreaKHis dataset	
Ak et al., 2020 [21]	LR	A comparative analysis of breast cancer detection and diagnosis using data visualization and machine learning applications.	98.1%
Sharma and Mishra et al., 2022 [22]	Voting classifier	Performance analysis of machine learning based optimized feature selection approaches for breast cancer diagnosis.	99.41%
Binsaif N et al., 2022 [23]	RF	Application of Machine Learning Models to the Detection of Breast Cancer.	83.3%

Our contributions include (i) the use of machine learning algorithms instead of traditional statistical regression methods to process clinical characterization data of patients with IDC to identify patients at high risk of cancer metastasis. (ii) This paper compares several algorithms that can be used to process patient data and determines that a fusion model based on a voting mechanism is the best way to process the data. (iii) It provides a scientific basis and reference for doctors to formulate personalized diagnosis and treatment plans and decisions for patients with different survival prediction results, thus improving treatment outcomes; (iv) It provides a useful reference for the formulation and modification of medical policies.

## 2 System model and methods

### 2.1 Data acquisition

**Fig 1:**
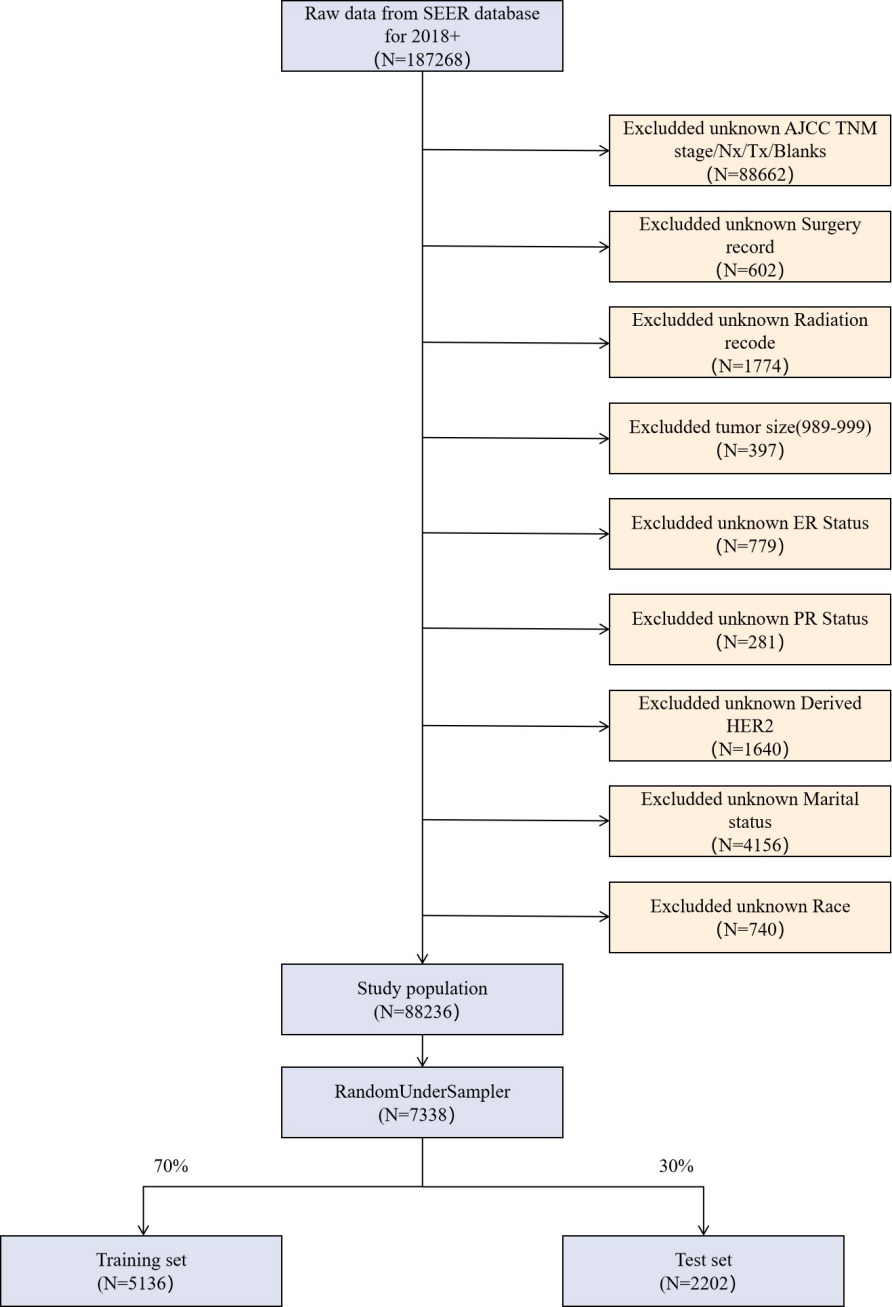
Data inclusion and exclusion process.

The data used in this experiment were obtained using SEER*stat software (version 8.4.0.1, National Cancer Institute, United States) based on the Surveillance, Epidemiology, and End results (SEER) database, using the "International Classification of Oncological Diseases, Third Edition (ICD-O-3).” The inclusion criteria were (Fig 1): (1) tumor location: site recode ICD-O-3 for breast; (2) pathologic tissue ICD-O-3 staging of 8500/3 (invasive ductal carcinoma); (3) tumor staging using the 8th edition of the American Joint Committee of Cancer (AJCC) TNM staging criteria; (4) availability of complete clinical, pathologic and follow-up data. The exclusion criteria were: (1) missing or unknown disease-related information; (2) age <28 years or >75 years; (3) unknown information such as T, N, and M staging, race, grading, marital status, chemotherapy status, and radiotherapy status; and (4) incomplete follow-up information.

### 2.2 Data pre-processing

Data mining and analysis in this study were done by Python (version 3.10, Python Software Foundation) and SPSS (version 23, IBM, USA). For data preprocessing: (1) Read the dataset: after screening the dataset, there were no missing, abnormal, or duplicated data; (2) Since the positive and negative samples were extremely unbalanced among the included ending variables, this study chose to introduce the function RandomUnderSampler() used to randomly undersample the data from the imblearn library, and resample the data so that the positive to negative sample ratio was 1:1; (3) The categorical feature variables are dummy coded and converted into dummy variables; (4) The data are randomly split into training and test sets in a ratio of 7:3 using train_test_split() in scikit-learn; (5) The transformed dataset is normalized using StandardScaler() to remove the inter-variable scale relationships; (6) Univariate analyses were performed to compare variables between patients who had or had not developed cancer metastases. For categorical data, the chi-square test was used; for continuous non-normally distributed data, the Wilcoxon rank sum test was used. Multivariate logistic regression was performed for variables with P < 0.05 in univariate analysis to determine which variables were strongly associated with the development of metastases. Effective data preprocessing is the prerequisite basis for model building and training.

### 2.3 Model establishment

**Fig 2:**
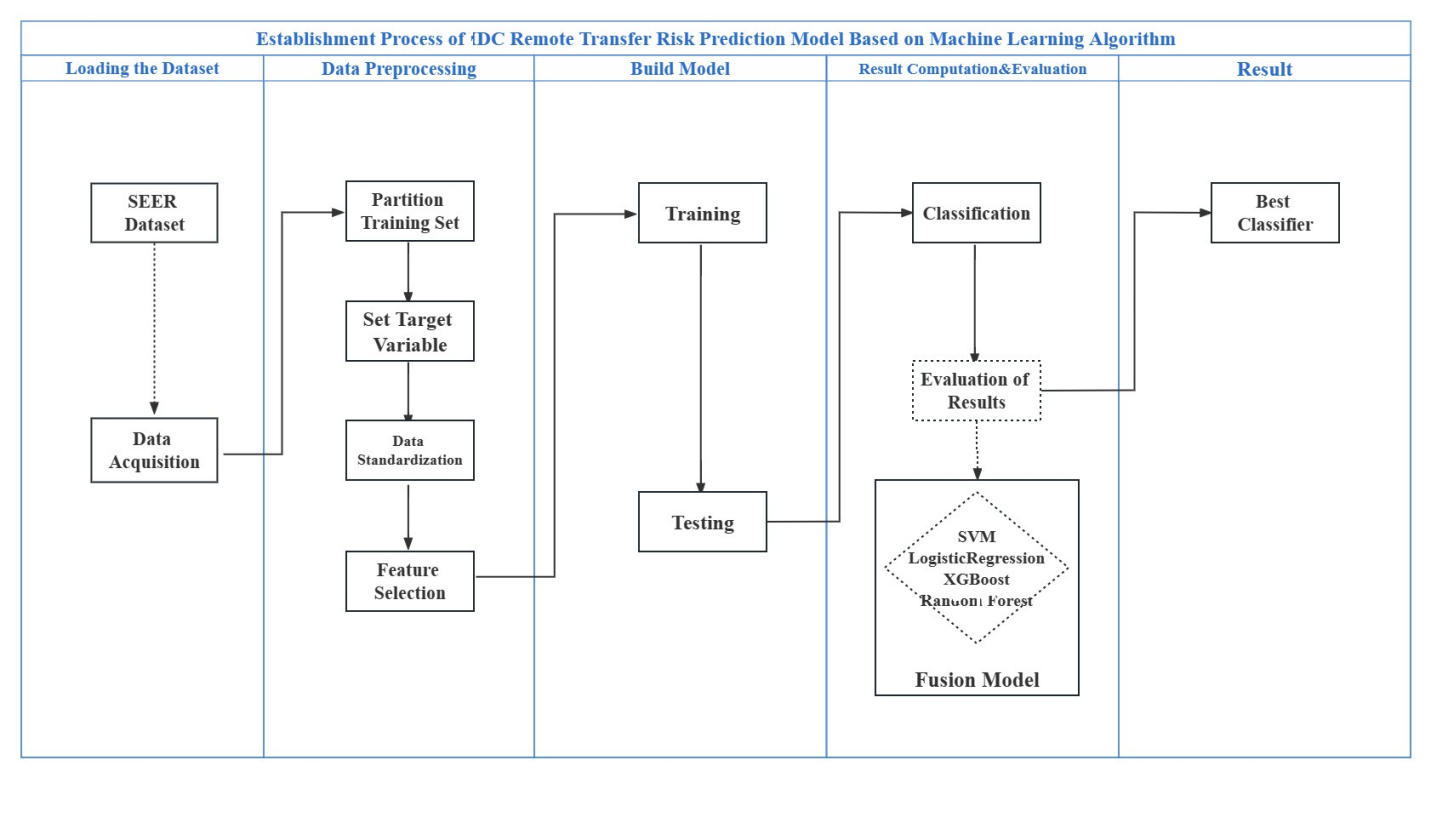
Modeling flowchart.

The training and testing procedures of the machine learning models were demonstrated in the JupyterNotebook environment using Python. Python sklearn and pandas software packages were used for data analysis, and Python matplotlib and seaborne were used to draw graphics. The basic modelling process is illustrated in Fig 2. The experimental environment configuration and the details of the software packages used for model development are shown in Tables 2 and 3, and the list of abbreviations is shown in Table 4.

**Table 2:** Experimental environment configuration table.

Hardware Environment	Software Environment
CPU Inter Corel i5–6300hq	Windows 10
GPU GTX960m	Python 3.10
RAM 8GB	PyCharm2021.3.3

**Table 3:** Detailed information on software packages used for machine learning model development.

Package name	Version	Description
Numpy	1.25.0	Numpy is the fundamental package for array computing with python
Pandas	2.1.0	Powerful data structures for data analysis, time series, and statistics
Matplotlib	3.5.1	Python plotting package
Sklearn	0.22.0	A set of python modules for machine learning and data mining
Imblearn	0.11.0	Toolbox for imbalanced dataset in machine learning
Seaborn	0.12.2	It provides a high-level interface for drawing attractive and informative statistical graphics.
XGBoost	1.2.0	XGBoost python package

**Table 4:** List of abbreviations.

Full noun name	Abbreviation
Accuracy	ACC
Area under ROC curve	AUC
Logistic Regression	LR
Support Vector Machine	SVM
EXtreme Gradient Boosting	XGBoost
Invasive Ductal Carcinoma	IDC
Random Forest	RF
Machine Learning	ML

Support Vector Machine (SVM) is a supervised learning algorithm whose basic model is a maximally spaced linear classifier defined on the feature space, and whose basic idea is to maximize the marginal distance between the decision hyperplane and the instances closest to the boundary, with the aim of finding the optimal hyperplane that distinguishes between two different classes [24, 25].

Logistic Regression (LR) is used to predict the probability of an event occurring by establishing a logistic function (Eq 1) relationship between two or more predictor variables and an outcome variable, i.e., fitting the data to the logistic function. The Sigmoid function introduces a nonlinear element, so it can deal with 0/1 classification problems, and is often used to predict and discriminate the likelihood of a category or event [26, 27].


$$f(z)=\frac{1}{1+e^{-2}}$$ (1)

Random Forest (RF) is a bagging integrated learning algorithm that uses a randomly selected subset of training samples and variables to generate multiple decision trees, each of which is a classifier that produces a classification result. By forming a large number of trees into a random forest, the risk of overfitting is reduced and the error converges to some generalized value [4, 28, 29]. The pseudo code of the machine learning model is shown in Table 5, and the basic learner parameters are shown in Table 6.

**Table 5:** Pseudocode for machine learning models.

Algorithm: Logistic Regression
Step1: For i = 1 to n.
Step2: For each data instance, calculate the regression value.
Step3: Apply the Sigmoid function to each of obtained regression calculated values.
Step4: Finalize the class labels and weights.
Step5: Classify, If (*P*>0.5) assign label ‘1’otherwise, assign label ‘0’.
Algorithm:Linear SVM
Input: Data samples {x_i_,y_i_}, i = 1,2,. . .,n, where x_i_∈R^d^, y_i_∈{-1,+1}.
Output: Parameters ω*, b* and categorical decision function of the categorical hyperplane.
Step1: Construct the objective optimization function: $minimize\;L=\sum_{ij}\alpha_{i}\alpha_{j}y_{i}y_{j}x_{i}^{T}x_{j}-\sum_{i=1}^{n}\alpha_{i}$ $s.t=\sum_{i=1}^{n}\alpha_{i}y_{i}=1$ α_i_ ≥ 0 (i = 1,2,…,n)
Step2: Find the value of the α-vector with the smallest value of the objective optimization function α* vector;
Step3: Calculate the corresponding coefficients ω*. $\omega^{*}=\sum_{i=1}^{n}{\alpha^{*}x}_{i}y_{i}$
Step4: Find all S sample points that satisfy α_s_ > 0 {x_s_, y_s_}, i.e., support vectors, and compute the corresponding parameters b_s_* and all b_s_* of all bs and the average value of all b_s_* b*. $b_{s}^{*}=y_{s}-\sum_{n=1}^{n}\alpha_{i}x_{i}^{T}y_{i}x_{s}$ $b^{*}=\frac{1}{s}\sum_{i=1}^{s}b_{i}^{*}$
Step5: Output categorical decision function f(x). $f(x)=sgn(\omega^{*}x+b^{*})$
Algorithm: Random Forest
Input: The data sample training set S = {x_i_, y_i_}, i = 1,2,. . ., n, where x_i_∈R^d^, y_i_∈ {-1,+1}, and the number of iterations of the base classifier T.
Output: The final strong classifier f(x).
Step1: Perform bootstrap sampling on the original training set S to generate a new training set S_i_;
Step2: Randomly extract a portion of features from S_i_ and train the t-th decision tree model. Each tree has no pruning process and grows to the specified tree depth;
Step3: The category with the highest number of votes from the T base classifiers is voted as the final category of the sample to be tested.

**Table 6:** Base learner parameters.

Model name	parameter
Random Forest	N_jobs = 1
	N_estimators = 500
	Max_depth = 6
	Min_samples_leaf = 2
	Max_features = ‘sqrt’
	Verbose = 0
SVM	Kernel = ‘linear’
	C = 0.025
XGBoost	N_estimators = 2000
	Max_depth = 4
	Min_child_weight = 2
	Gamma = 0.9subsample = 0.8
	Objective = ‘binary:logistic’
	Scale_pos_weight = 1
Logistic Regression	N_jobs = 1
	penalty = ‘12’
	C = 1
	fit_intercept = True
	intercept_scaling = 1
	max_iter = 100

EXtreme Gradient Boosting (XGB) is a boosting integrated learning algorithm that is a gradient lifting algorithm based on the CART regression tree. After multiple iterations, each iteration produces a weak classifier, and each classifier is trained based on the gradient of the previous classifier round. The basic idea can be understood as taking the second-order Taylor expansion of the loss function as its replacement function. The objective function of the algorithm is composed of the loss function L of the model and the regular term Ω to restrain the complexity of the model.

**Fig 3:**
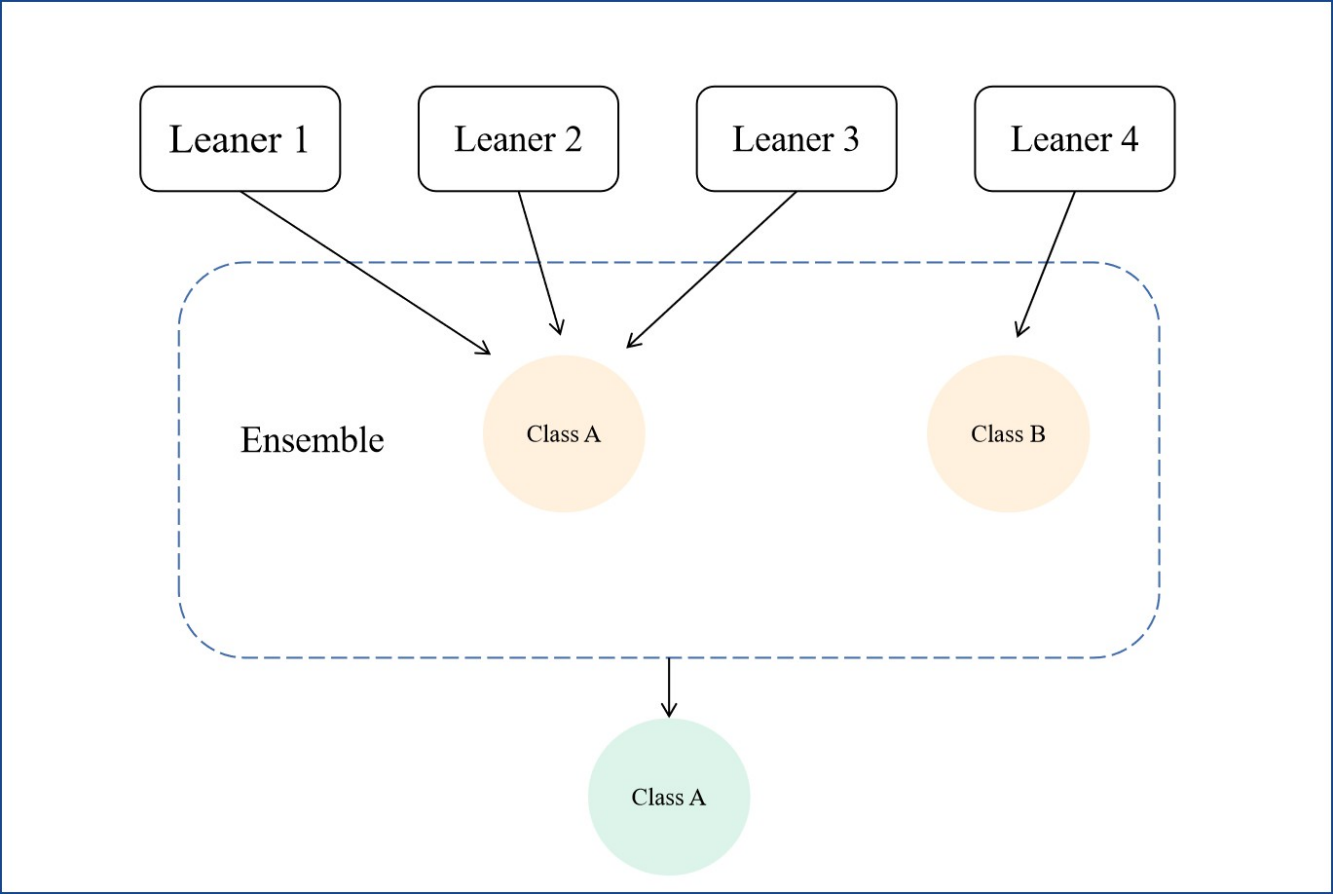
Schematic diagram of fusion model based on voting mechanism.

The four modeling algorithms mentioned above are built into a fusion model based on the voting mechanism, which is an integrated learning model following the principle of minority-majority, and reduces the variance through the integration of multiple models, thus improving the robustness of the model. Typically in the categorization case, the integrated learner *h*_*i*_*(x)* needs to predict a category token from the category labels, and in general, the frequently used method is the voting method (Eq 2). If more than half of the votes are cast for a token, it is expected to be that token; otherwise, the prediction is rejected. Theoretically the final output is the most popular prediction in the base learner. The basic principle is shown in Fig 3.


$$H(x)=\left\{ \begin{array}{c} c_{i},if\sum_{i=1}^{T}h_{j}^{i}(x)>0.5\sum_{k=1}^{N}\sum_{i=1}^{T}h_{j}^{k}(x)\\ reject,otherwise \end{array}\right.$$ (2)

### 2.4 Evaluation indicators

In artificial intelligence, the Confusion Matrix is a common visualization tool in supervised learning. Its main function is to summarize the results of a classifier. The common evaluation criteria used in evaluating the classification performance are: Accuracy, Precision, Recall, F1-score and the ROC curve (Eqs 3–7).


$$Accuracy=\frac{TP+TN}{TP+FN+FP+TN}$$ (3)

$$Precision=\frac{TP}{TP+FP}$$ (4)

$$Recall=\frac{TP}{TP+FN}$$ (5)

$$F1-score=\frac{2*Precision*Recall}{Precision+Recall}$$ (6)

$$AUC=\frac{\sum R_{i}(I_{p})-I_{p}(I_{p}+1)/2}{I_{p}+I_{n}}$$ (7)

Where TP refers to True Positive, TN refers to True Negative, FP refers to False Positive, and FN refers to False Negative; the Fl-score is a statistical measure of the precision of a dichotomous model, considering both the precision and the recall of the model. A higher F1 score indicates better performance of the model; AUC: The area under the curve is a numerical indicating how well the model will perform in different situations. In general, an AUC greater than 0.85 indicates excellent model performance; Learning Curve is a curve about the sample size of the training datasets, the average score on the training samples and cross-validation samples as well as between the score intervals. By observing the plotted Learning Curve graph, you gain a more intuitive understanding of the state of the model, such as: overfitting or underfitting.

## 3 Result

### 3.1 Demographic characterization

A total of 88,236 patients with confirmed IDC were included in this study from the SEER database, with 14 characteristic variables and outcome variables related to clinicopathology and patient demographics. Demographic variables included race, gender, marital status, and age, while clinicopathological variables included tumor size, estrogen status, progesterone status, HER2 status, TN staging, radiotherapy, and surgical treatment. All data distributions are shown in Table 7. The number of positive and negative samples was equalized by undersampling M1 (3669): M0 (3669) = 1:1. The entire SEER dataset was divided into a training set (n = 5136, 70%) and a test set (n = 2202, 30%). Detailed distribution information is shown in Table 8.

**Table 7:** Clinical and pathological characteristics of the study subjects.

Variables	All	M1	M0
	N = 88236	N = 3669	N = 84567
**Age**			
<50	19294 (21.9%)	914 (4.7%)	18380 (95.3%)
>50	68942 (78.1%)	2755 (3.9%)	66187 (96.0%)
**Sex**			
Female	87458 (99.1%)	3621 (4.1%)	83837 (95.8%)
Male	778 (0.8%)	48 (6.1%)	730 (93.8%)
**Race**			
White	68474 (77.6%)	2703 (3.9%)	65771 (96.0%)
Black	9486 (10.7%)	581 (6.1%)	8905 (93.8%)
American Indian/Alaska Native	575 (0.6%)	24 (4.1%)	551 (95.8%)
Asian or Pacific Islander	9701 (10.9%)	361 (3.7%)	9340 (96.2%)
**ER Status Recode**			
Negative	14662 (16.6%)	923 (6.2%)	13739 (93.7%)
Positive	73574 (83.3%)	2746 (3.7%)	70828 (96.2%)
**PR Status Recode**			
Negative	23771 (26.9%)	1359 (5.8%)	22376 (94.1%)
Positive	64465 (73.0%)	2274 (3.5%)	62191 (96.4%)
**HER2 Recode**			
Negative	75169 (85.1%)	2779 (3.6%)	72390 (96.3%)
Positive	13067 (14.8%)	890 (6.8%)	12177 (93.1%)
**Marital status**			
Single	14303 (16.4%)	13493 (94.3%)	810 (5.6%)
Married	51439 (58.3%)	1768 (3.4%)	49671 (96.5%)
Divorced	9740 (11.0%)	461 (4.7%)	9279 (95.2%)
Unmarried or Domestic Partner	576 (0.7%)	550 (95.4%)	26 (4.5%)
Widowed	11203 (12.6%)	545 (4.8%)	10658 (95.1%)
**Tumor Size(mm)**			
<100	87597 (99.3%)	3457 (3.9%)	84140 (96.0%)
>100	639 (0.7%)	212 (33.1%)	427 (66.8%)
**Chemotherapy recode**			
Yes	34839 (39.4%)	2722 (7.8%)	32117 (92.1%)
None	53397 (60.5%)	947 (1.7%)	52450 (98.2%)
**Radiation recode**			
Yes	50357 (57.1%)	1245 (2.4%)	49112 (97.5%)
None	37879 (42.9%)	2424 (6.3%)	35455 (93.6%)
**Surgery recode**			
Surgery performed	83233 (94.3%)	1144 (1.3%)	82089 (98.6%)
Not performed	5003 (5.6%)	2525 (50.4%)	2478 (49.5%)
**T stage**			
T0	110 (0.1%)	30 (27.2%)	80 (72.7%)
T1	54376 (61.6%)	618 (1.1%)	53758 (98.8%)
T2	26813 (30.3%)	1361 (5.0%)	25452 (94.9%)
T3	4277 (4.8%)	648 (5.1%)	3629 (84.8%)
T4	2660 (3.0%)	1012 (38.0%)	1648 (61.9%)
**N stage**			
N0	62875 (71.3%)	905 (1.4%)	61970 (98.5%)
N1	20359 (23.1%)	1688 (9.1%)	18493 (90.8%)
N2	2741 (3.1%)	307 (11.2%)	2434 (88.7%)
N3	2261 (2.6%)	591 (26.1%)	1670 (73.8%)

**Table 8:** Distribution of clinical pathological features in training and testing sets.

Variables	Training set (N = 5136)		Test set (N = 2202)	
	M0 (N = 2584)	M1 (N = 2552)	M0 (N = 1085)	M1 (N = 1117)
**Age**				
<50	573	643 (25.2%)	215 (19.8%)	217 (24.3%)
>50	2011	1909 (74.8%)	870 (80.2%)	846 (75.7%)
**Sex**				
Male	22 (0.8%)	31 (1.2%)	5 (0.4%)	17 (1.5%)
Female	2562 (99.1%)	2521 (98.7%)	1080 (99.5%)	1100 (98.4%)
**Race**				
White	1978 (76.5%)	410 (16.0%)	840 (77.4%)	828 (74.1%)
Black	277 (10.7%)	1875 (73.4%)	130 (11.9%)	171 (15.3%)
American Indian/Alaska Native	24 (0.9%)	15 (0.6%)	4 (0.4%)	9 (10.8%)
Asian or Pacific Islander	305 (11.8%)	252 (9.8%)	111 (10.2%)	109 (9.7%)
**ER Status Recode**				
Negative	410 (15.8%)	655 (25.6%)	168 (15.4%)	268 (23.9%)
Positive	2174 (84.1%)	1897 (74.3%)	917 (84.5%)	849 (76.0%)
**PR Status Recode**				
Negative	712 (27.5%)	975 (38.2%)	288 (26.5%)	420 (37.6%)
Positive	1872 (72.4%)	1577 (61.7%)	797 (73.4%)	697 (62.3%)
**HER2 Recode**				
Negative	2197 (85.0%)	1918 (75.1%)	941 (86.7%)	861 (77.0%)
Positive	387 (14.9%)	634 (24.8%)	144 (13.2%)	256 (22.9%)
**Marital status**				
Married	1522 (58.9%)	1239 (48.6%)	624 (57.5%)	529 (47.4%)
Single	400 (15.4%)	557 (21.8%)	165 (15.2%)	253 (22.6%)
Divorced	271 (10.4%)	325 (12.7%)	123 (11.3%)	136 (12.1%)
Unmarried or Domestic Partner	20 (0.8%)	18 (0.7%)	5 (0.5%)	8 (0.7%)
Widowed	331 (12.8%)	369 (14.4%)	154 (14.1%)	176 (15.7%)
Separated	40 (1.5%)	44 (1.7%)	14 (1.2%)	15 (1.3%)
**Tumor Size(mm)**				
<100	2571 (99.5%)	2403 (94.2%)	1083 (99.8%)	1052 (94.2%)
>100	13 (0.5%)	149 (5.8%)	2 (0.2%)	65 (5.8%)
**Chemotherapy recode**				
None	1594 (61.6%)	656 (25.7%)	393 (36.2%)	291 (26.0%)
Yes	990 (38.3%)	1898 (74.2%)	692 (63.7%)	826 (73.9%)
**Radiation recode**				
None	1097 (42.5%)	1702 (66.7%)	481 (44.3%)	722 (64.6%)
Yes	1487 (57.5%)	850 (33.3%)	604 (55.7%)	395 (35.4%)
**Surgery recode**				
None	79 (3.1%)	1739 (68.1%)	41 (3.8%)	786 (70.4%)
Yes	2505 (96.9%)	813 (31.8%)	1044 (96.2%)	331 (29.6%)
**T stage**				
T0	1 (<0.1%)	17 (0.6%)	1 (<0.1%)	13 (1.1%)
T1	1628 (63.0%)	434 (17.0%)	703 (64.8%)	184 (16.5%)
T2	779 (30.1%)	940 (36.8%)	313 (28.8%)	421 (37.6%)
T3	113 (4.4%)	451 (17.6%)	43 (3.9%)	197 (17.6%)
T4	63 (2.4%)	710 (27.8%)	25 (2.3%)	302 (27.0%)
**N stage**				
N0	1916 (74.1%)	628 (24.6%)	813 (74.9%)	277 (24.8%)
N1	546 (21.1%)	1291 (50.6%)	225 (20.7%)	575 (51.5%)
N2	73 (2.8%)	220 (18.6%)	24 (2.2%)	87 (7.8%)
N3	49 (1.9%)	413 (16.2%)	23 (2.1%)	178 (15.9%)

### 3.2 One-way logistic regression analysis

Univariate chi-square test analysis showed (Table 9) that age, gender, race, ER/PR/HER2 status, marital status, tumor size, radiological and surgical treatments, and TN staging in IDC patients were significantly associated with the outcome variable M (P<0.05). Additionally, one-way logistic regression analysis of the significantly different variables showed that HER2 status, tumor size, radiotherapy, chemotherapy, surgical treatment, and TN staging may be risk factors for the development of cancer metastasis in patients with IDC. These six variables were used for further machine learning modeling studies.

**Table 9:** Univariate and multivariate logistic regression analysis.

Variables	Univariate analysis		Multivariate logistic regression analysis	
	Chi-Square	P-value	OR (95%CI)	P-value
**Age**	125.398	<0.001		
<50			Reference	
>50			1.031 (0.865–1.229)	0.743
**Sex**	5.941	0.015		
Male			Reference	
Female			1.802 (0.947–3.429)	0.073
**Race**	37.240	<0.001		
White			Reference	
Black			1.285 (0.558–2.962)	0.556
American Indian/Alaska Native			1.153 (0.504–2.639)	0.736
Asian or Pacific Islander			1.560 (0.695–3.501)	0.281
**ER Status Recode**	99.689	<0.001		
Negative			Reference	
Positive			0.943 (0.744–1.195)	0.627
**PR Status Recode**	96.711	<0.001		
Negative			Reference	
Positive			1.178 (0.956–1.451)	0.123
**HER2 Recode**	112.479	<0.001		
Negative			Reference	
Positive			1.205 (1.000–1.451)	0.050
**Marital status**	89.147	<0.001		
Married			Reference	
Single			0.851 (0.672–1.078)	0.182
Divorced			0.868 (0.454–1.660)	0.669
Unmarried or Domestic Partner			0.993 (0.755–1.306)	0.959
Separated			0.899 (0.667–1.212)	0.484
Widowed			0.910 (0.317–2.087)	0.825
**Tumor Size(mm)**	2186.407	<0.001		
<100			Reference	
>100			2.513 (1.360–4.644)	0.003
**Chemotherapy recode**	991.334	<0.001		
Yes			Reference	
None			2.343 (1.960–2.799)	<0.001
**Radiation recode**	393.383	<0.001		
Yes			Reference	
None			0.635 (0.546–0.740)	<0.001
**Surgery recode**	3419.256	<0.001		
Yes			Reference	
None			0.027 (0.022–0.033)	<0.001
**T stage**	2126.272	<0.001		
T0			Reference	
T1			0.155 (0.031–0.785)	0.897
T2			0.277 (0.055–1.401)	0.430
T3			0.518 (0.101–2.652)	0.121
T4			1.115 (0.217–5.736)	0.024
**N stage**	1885.641	<0.001		
N0			Reference	
N1			2.419 (2.039–2.871)	<0.001
N2			4.238 (3.105–5.783)	<0.001
N3			8.785 (6.424–12.015) (6.424–12.015)	<0.001

### 3.3 Correlation analysis of characteristic variables

**Fig 4:**
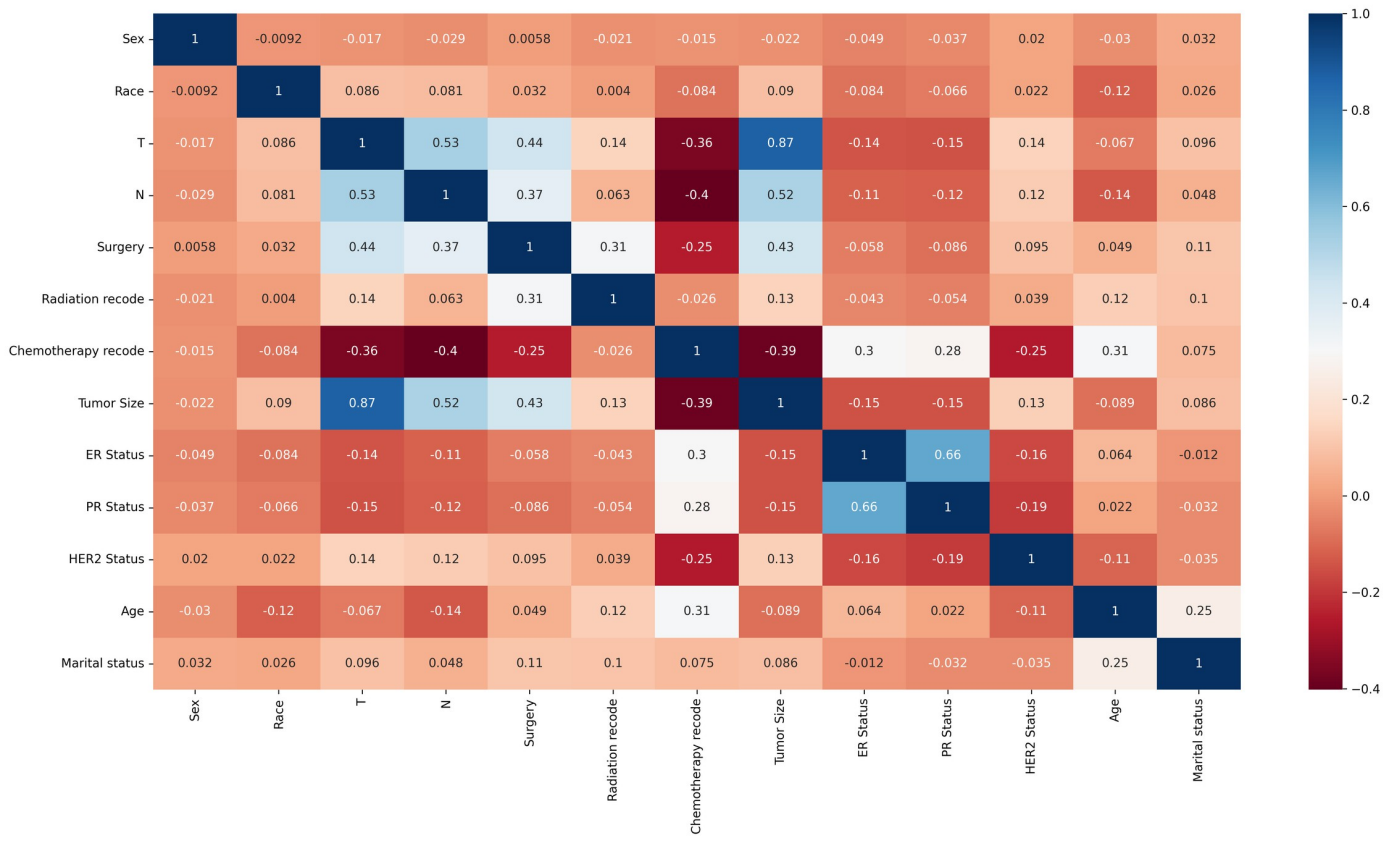
Heat map of the correlation of features.

The correlation analysis of the feature variables of the datasets demonstrates information about the degree of interaction among these variables. This study uses heat maps to show the correlation of these features with the predictions of the ML algorithm. In the heat map, color indicates the degree of correlation, the darker the color the higher the correlation between the parameters. Fig 4 shows the correlation matrix of 13 variables in the datasets, displaying the correlation between variables. Each cell in the matrix shows the Spearman correlation coefficient between two variables. From the graph, it can be seen that there is a significant negative correlation between chemotherapy and TN staging, while T staging is significantly positively correlated with tumor size. In clinical practice, the definition of T staging is based on the diameter of the tumor. This is consistent with clinical experience.

### 3.4 Model performance evaluation

**Fig 5:**
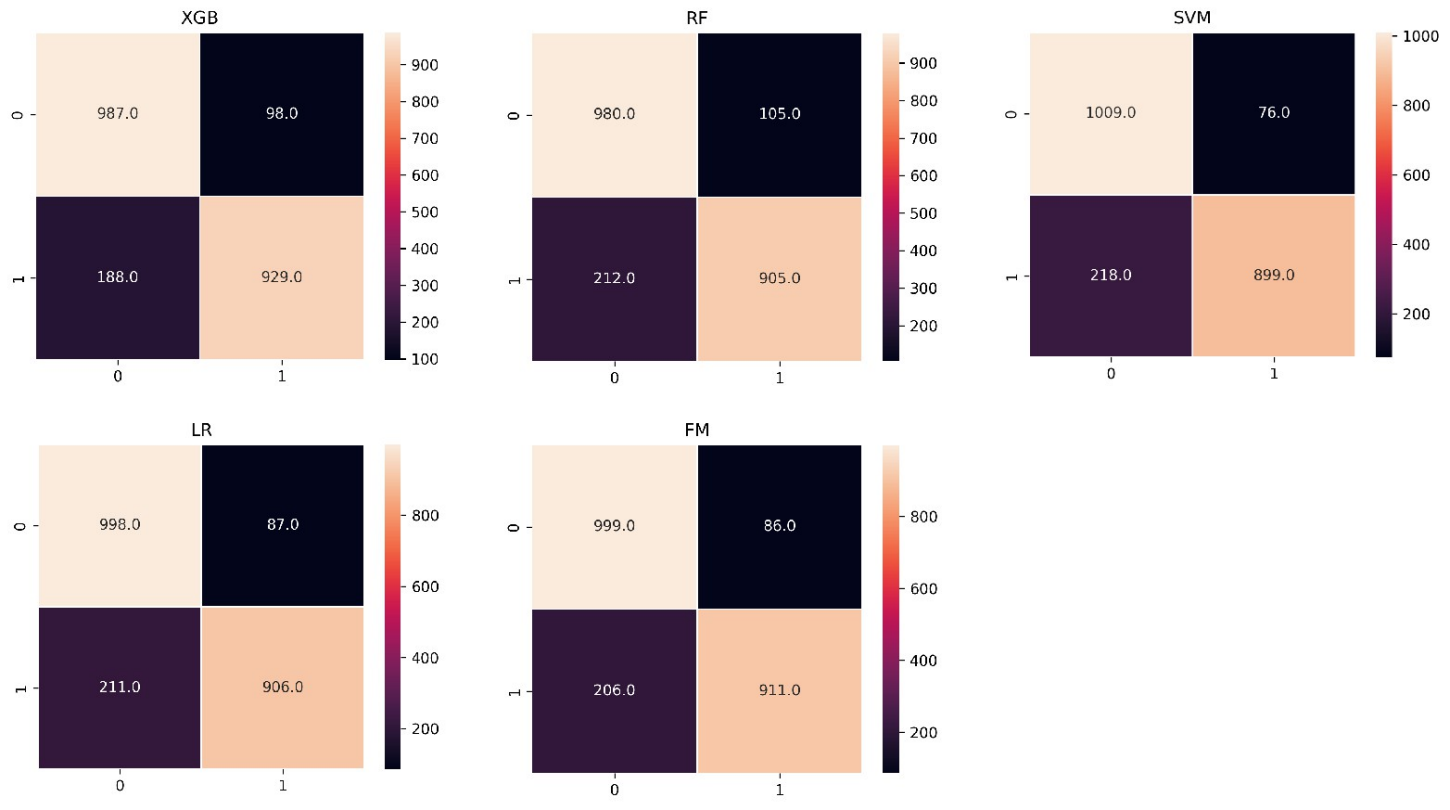
The confusion matrix of the models in the test set.

**Fig 6:**
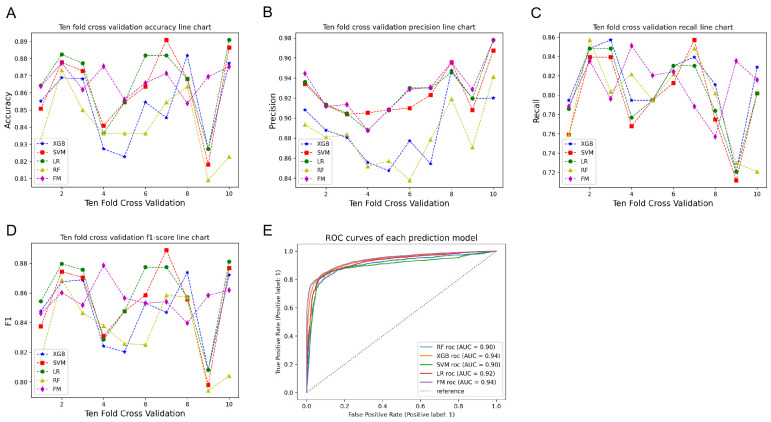
Comparison of Ten-fold Cross validation metrics for various machine learning models.

**Fig 7:**
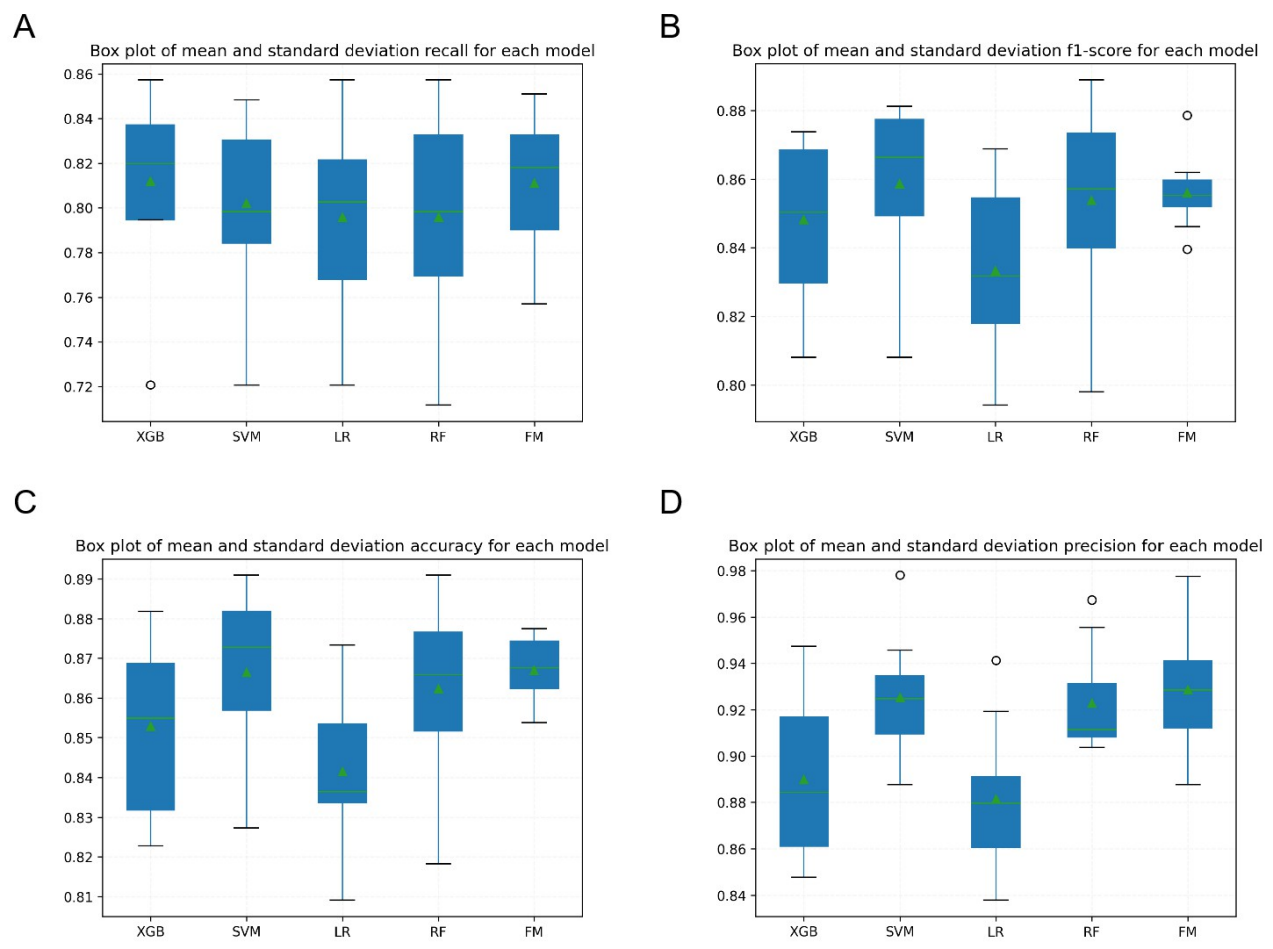
Box plot comparing the average and standard deviation of indicators.

**Fig 8:**
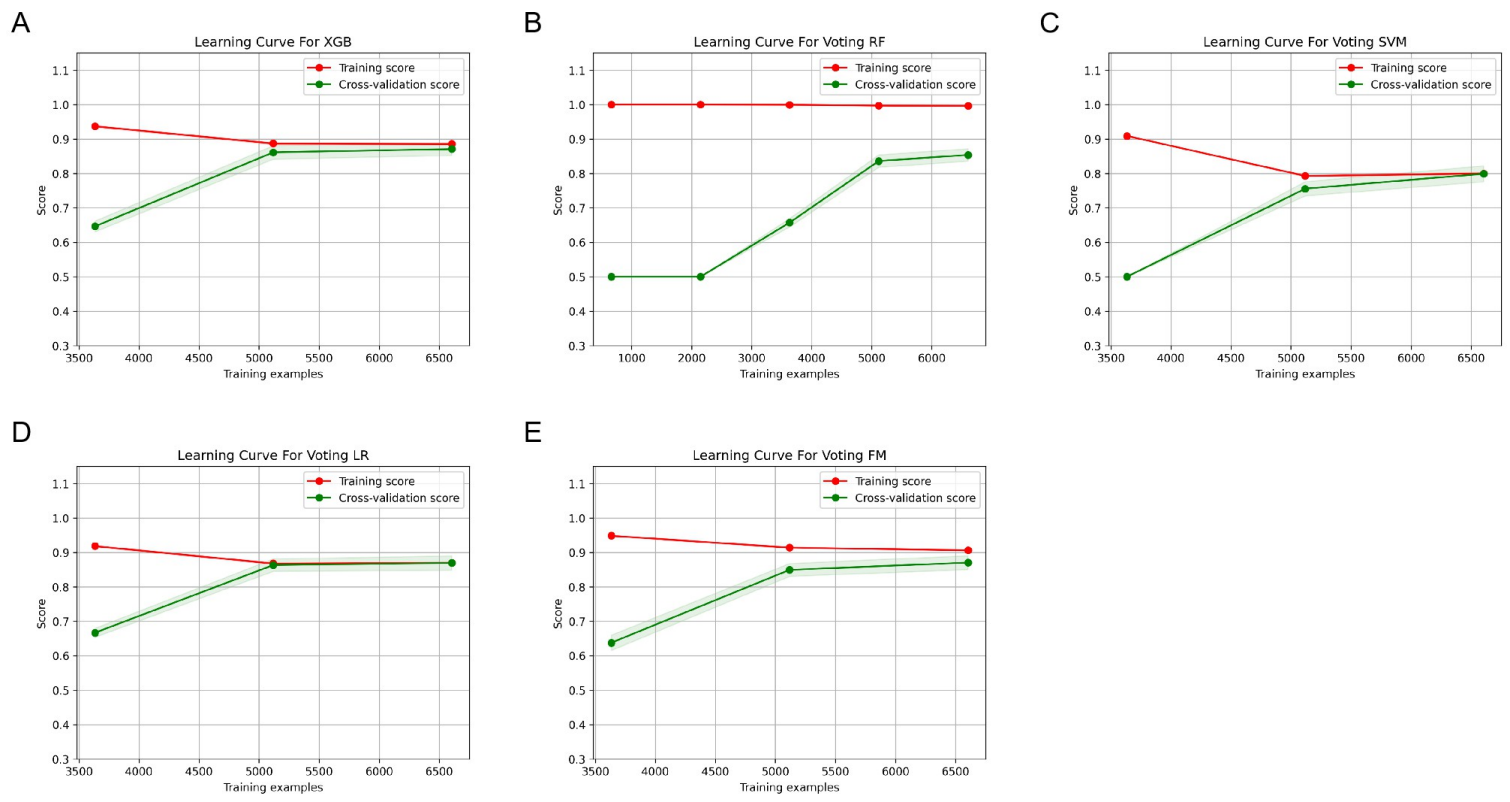
The learning curve of each machine learning prediction model.

The performance of six machine models was built and compared based on the confusion matrix, learning curves, receiver operating characteristic curves (ROCs), ten-fold cross-validation accuracy, precision, recall, F1-score and AUC value. The results are shown in Figs 5–8. Each model has its relative advantages. Taken together, the fusion model has higher indicators, with accuracy, Recall, Precision, F1-score, and AUC values of 0.867, 0.805, 0.929, 0.856, and 0.94, respectively. One of the biggest drawbacks of the fusion model based on voting mechanism is that if our fusion algorithms are all carefully tuned algorithms, the voting scheme may lead to overfitting. The assessment of the model state was performed using learning curves, and after cross-validation, the fitting effect of RF and SVM is very unsatisfactory. The LR model is the best. Although FM is not the best, it has already reached a relatively ideal state.

### 3.5 Importance analysis of characteristic variables

**Fig 9:**
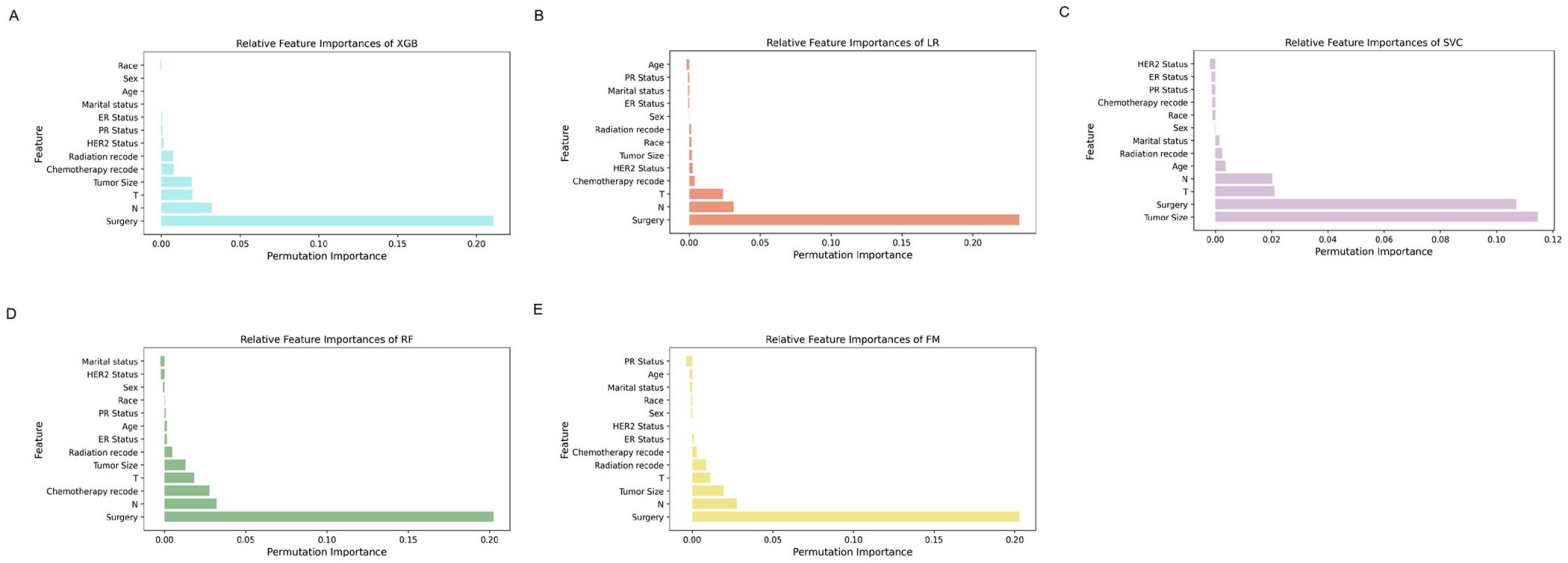
Contributions of the clinical features variables to risk prediction using feature importance.

The PermutationImportance method provided by eli5 in Python is used to calculate the importance of features. Fig 9 shows the feature importance ranking of the five prediction models for cancer metastasis prediction for the 13 feature variables after dimensionality reduction processing on the training set. Variables screened by univariate and multivariate logistic analyses contributed to the predictions in all five models. Surgery ranked first or second in terms of feature importance among all predictive models, indicating that surgery has a strong impact on the prediction of cancer metastasis in IDC. Variables such as age, gender, and marital status always rank in the bottom five, and there is no significant difference in their contribution to the model.

## 4 Discussion

Over the past few decades, the incidence of death in patients with BC has decreased significantly due to advances in surgical methods and systematic neoadjuvant chemotherapy [30]. Metastatic BC, rather than primary tumors, causes more than 90% of cancer-related deaths [31]. As there are no significant symptoms in the early stage of BC, it is easy to be ignored by patients. As a result, the disease has often developed to the middle and late stages when it is found, and recurrence and metastasis are easy to occur after systematic treatment, which is not only difficult to treat, but also greatly affects the quality of life of the patients. In the current treatment process, pathological examination is the gold standard for metastatic tumors. When a physician suspects that a patient has metastatic cancer somewhere, they usually first have the patient undergo a series of immunohistochemical tests to aid in the diagnosis; not only will this invasive test be more physically harmful to the patient, but the pathology results are usually available 2 to 3 days after the test. This waiting time is critical to improving the survival chances of patients with advanced hematogenous metastatic BC [32]. Therefore, early identification of patients with IDC at risk of developing distant metastases from cancer is extremely important. With the rapid advancements in computer technology and artificial intelligence, it has become a trend to use machine learning to analyze patient data to help doctors make correct judgments [33]. Currently, related scholars have achieved promising results in the prediction of survival rate and survival status of cancer patients using machine learning methods such as random forest, SVM, and neural network [34, 35].

This study uses only clinical and pathological indicators of patients with IDC obtained based on the SEER database to establish a validated prediction model. This model aims to classify and predict whether a patient has cancer metastasis, which can easily predict the risk of organ metastasis without imaging or any laboratory tests. This model can help clinicians make a rapid and reliable diagnosis and improve their work efficiency, and can develop individualized treatment plans for different individuals [36], saving treatment time for patients by identifying the risk of metastasis early, thus reducing mortality.

This study builds a prediction model based on four algorithms: logistic regression, SVM, random forest, and XGBoost. From the results after ten-fold cross-validation, each prediction model has its own advantages and disadvantages. The accuracy and AUC of the XGBoost model predictions are relatively high, which indicates a high degree of overall prediction accuracy, along with the high ability of this classifier to rank samples. However, the precision, recall and F1 value of this model are low, which indicates its low prediction accuracy in positive sample results, and a potential for underdiagnosis. Conversely, the LR and RF models have low metrics and are not suitable as good classifiers for risk prediction. The performance of the fusion model based on the voting mechanism on all the indicators is improved to a certain extent compared to the individual base models. High accuracy indicates that the model can classify patients more accurately, high sensitivity implies a low rate of missed diagnosis, and low error indicates stable predictive performance of the model. In addition, learning curves were used to diagnose the overfitting behavior of the model. As the number of training samples increased, the accuracy gap between RF on the training and cross-validation sets was large, indicating that the models can fit the known data well, but the generalization ability is very poor, which is an overfitting. The learning curves of other models can converge on both the training and validation sets, but SVM is only 0.75, with a slight deviation and unsatisfactory fitting. At this time, FM convergence value was approaching 0.9, indicating that the algorithm itself has good fitting ability, the model’s generalization ability is also very strong, and the fitting effect is price ideal. Therefore, the Fusion classifier is more suitable as a good predictive model to predict the risk of IDC distant organ transfer. This study is innovative in using ML algorithms to predict the risk of cancer metastasis in patients with IDC. Currently, a number of academics and researchers in the medical field have used traditional linear statistical models and a single machine learning model to predict the risk of BM in patients with IDC [37–39], but few studies have used multi-model fusion techniques for modeling and prediction. In this study, a fusion machine learning model based on multiple algorithms was developed, which was able to more accurately predict the risk of cancer metastasis in patients with IDC and outperformed other single machine learning models developed in this study.

The ranking importance principle was used to analyze the relative characteristic importance of the variables in each ML model [40]. We found that although the relative feature importance varied slightly across ML models, Surgery, T, N staging, and Tumor Size were the top-ranked variables in most of the models, in contrast to the age, race and ER Status variables, which was the last-ranked variable in most of the models, but also contributed to the models. In this study, five variables, Surgery, T and N staging, Tumor Size, Chemotherapy record, were strong predictors of the risk of distant metastasis of IDC with good predictive value. This suggests that for which variables were crucial for the creation of the predictive model in this experiment, these characterizing variables could be considered for inclusion in the study when conducting such experiments in the future, to inform the selection of variables in the database.

In the field of medicine, many researchers have attempted to identify independent risk factors that influence the development of cancer metastasis in patients with IDC. In this study, we analyzed the pathological characteristic variables of the patients’ based on one-way logistic regression and delved into the risk factors affecting the development of cancer metastasis in the patients. We identified that HER2 status, tumor size, radiotherapy, chemotherapy, surgical treatment, and TN staging are risk factors for cancer metastasis in IDC. In clinical practice, patients with low and positive HER2 expression generally have higher tumor grades, larger tumors, more lymph node metastases [41], a smaller percentage of lobular carcinomas [42], and an increased risk of brain metastases [43]. Qi et al. obtained a higher risk of metastasis in HER2-positive patients than in HER2-negative patients in their analysis of risk factors for developing internal breast lymph node metastasis in BC [44], which is consistent with the results of our study. Tumor size was positively correlated with the odds of developing breast tumors. Yazdani et al. found that tumor size was a risk factor for developing bone metastases in BC, and the larger the tumor, the greater the likelihood of bone metastases [45]. In clinical practice, T stage means the size and extent of the primary tumor. In our results, patients with tumor diameters greater than 100 mm and T4 stage were more likely to develop metastasis of cancer cells, which is also consistent with the results of related studies and clinical experience. Poortmans et al [46] reported a lower rate of lymph node metastasis (<1.5%) in patients with BC receiving systemic therapy. In our results, patients receiving systemic therapy were less likely to develop cancer metastasis. The results of this study provide a theoretical basis for clinicians to assess the prognosis of patients with IDC, which can be used by physicians to develop more precise and individualized treatment plans for their patients.

However, few studies have focused on combining machine learning to predict the risk of distant metastasis of cancer cells in IDC. Therefore, in this study, we built a model with good predictive performance and good ability to fit the data for predicting the risk of cancer metastasis in IDC patients, based on data from the SEER database and incorporating four machine learning models. Our predictive model allows clinicians to easily input relevant clinical and pathological indicators of their patients into the model, which will calculate the patient’s risk of cancer metastasis and then make quick and accurate personalized decisions for their patients based on the predictive output, and can be used as the basis for clinicians to explain their decisions to patients and engage them in their treatment choices. However, our study has certain limitations. Firstly, the data used in this study were limited by the region, quantity, and subjectivity of the feature selection, which limited the final experimental results. Secondly, when artificial intelligence is integrated into the interpretation by clinical physicians, there may be diverse manifestations in clinical practice [47]. Moreover, this was a retrospective study that needs to be validated through prospective research.

## 5 Conclusion

In this study, we constructed an advanced prediction model that integrates four algorithms: random forest, logistic regression, SVM, and XGB, based on a voting mechanism. This risk prediction model has superior predictive performance and high medical application value. The integrated model based on the voting mechanism shows further improvement upon the already effective predictions of individual models. This enhancement contributes to the improved ability of physicians to accurately identify malignant tumors in breast cancer, holding practical significance for timely and effective treatment for breast cancer patients.

## Supporting information

S1 Data (ZIP)

## References

[1] Li L, Tian H, Zhang B, et al. Prediction for distant metastasis of breast cancer using dynamic contrast-enhanced magnetic resonance imaging images under deep learning[J]. Computational Intelligence and Neuroscience, 2022: 6126061. doi: 10.1155/2022/6126061 35720877 PMC9200535

[2] Balaha HM, Antar ER, Saafan MM, et al. A comprehensive framework towards segmenting and classifying breast cancer patients using deep learning and Aquila optimizer[J]. Journal of Ambient Intelligence and Humanized Computing, 2023, 14(6): 7897–7917.

[3] Sung H, Ferlay J, Siegel RL, et al. Global Cancer Statistics 2020: GLOBOCAN Estimates of Incidence and Mortality Worldwide for 36 Cancers in 185 Countries[J]. CA Cancer J Clin, 2021, 71(3): 209–249. 10.3322/caac.21660 33538338

[4] Bray F, Ferlay J, Soerjomataram I, et al. Global cancer statistics 2018: GLOBOCAN estimates of incidence and mortality worldwide for 36 cancers in 185 countries[J]. CA: a cancer journal for clinicians, 2018, 68(6): 394–424. doi: 10.3322/caac.21492 30207593

[5] Baghdadi NA, Malki A, Balaha HM, et al. Classification of breast cancer using a manta-ray foraging optimized transfer learning framework[J]. PeerJ Computer Science, 2022, 8: e1054. 10.7717/peerj-cs.1054 PMC9454783 36092017

[6] Böger C, Behrens HM, Röcken C. Ki67—An unsuitable marker of gastric cancer prognosis unmasks intratumoral heterogeneity[J]. J Surg Oncol, 2016, 113(1): 46–54. 26709194 10.1002/jso.24104 PMC4736456

[7] La Vecchia C, Bosetti C, Lucchini F, et al. Cancer mortality in Europe, 2000–2004 and an overview of trends since 1975[J]. Ann Oncol, 2010, 21(6): 1323–1360. 19948741 10.1093/annonc/mdp530

[8] Liu W, Zhang R, Ling Y, et al. Automatic recognition of breast invasive ductal carcinoma based on terahertz spectroscopy with wavelet packet transform and machine learning[J]. Biomedical optics express, 2020, 11(2): 971–981. 32206399 10.1364/BOE.381623 PMC7041450

[9] Ma W, Wang X, Xu G, et al. Distant metastasis prediction via a multi-feature fusion model in breast cancer[J]. Aging (Albany NY), 2020, 12(18): 18151. 32989175 10.18632/aging.103630 PMC7585122

[10] Dheeba J, Singh NA, Selvi ST. Computer-aided detection of breast cancer on mammograms: A swarm intelligence optimized wavelet neural network approach[J]. Journal of biomedical informatics, 2014, 49: 45–52. 24509074 10.1016/j.jbi.2014.01.010

[11] Naji MA, El Filali S, Aarika K, et al. Machine learning algorithms for breast cancer prediction and diagnosis[J]. Procedia Computer Science, 2021, 191: 487–492.

[12] Choi RY, Coyner AS, Kalpathy-Cramer J, et al. Introduction to Machine Learning, Neural Networks, and Deep Learning[J]. Transl Vis Sci Technol, 2020, 9(2): 14–15. 10.1167/tvst.9.2.14 PMC7347027 32704420

[13] Battineni G, Chintalapudi N, Amenta F. Performance analysis of different machine learning algorithms in breast cancer predictions[J]. EAI Endorsed Transactions on Pervasive Health and Technology, 2020, 6(23).

[14] Li J, Zhou Z, Dong J, et al. Predicting breast cancer 5-year survival using machine learning: A systematic review[J]. PloS one, 2021, 16(4): e0250370. 33861809 10.1371/journal.pone.0250370 PMC8051758

[15] Ahmad S, Ullah T, Ahmad I, et al. A novel hybrid deep learning model for metastatic cancer detection[J]. Computational Intelligence and Neuroscience, 2022, 2022(1): 8141530. 35785076 10.1155/2022/8141530 PMC9249449

[16] Islam M M, Haque M R, Iqbal H, et al. Breast cancer prediction: a comparative study using machine learning techniques[J]. SN Computer Science, 2020, 1: 1–14.

[17] Choudhury A. Predicting cancer using supervised machine learning: Mesothelioma[J]. Technology and Health Care, 2021, 29(1): 45–58. 32568137 10.3233/THC-202237

[18] Alfian G, Syafrudin M, Fahrurrozi I, et al. Predicting breast cancer from risk factors using SVM and extra-trees-based feature selection method[J]. Computers, 2022, 11(9): 136–137.

[19] Afolayan JO, Adebiyi MO, Arowolo MO, et al. Breast cancer detection using particle swarm optimization and decision tree machine learning technique[M]//Intelligent Healthcare: Infrastructure, Algorithms and Management[J]. Singapore: Springer Nature Singapore, 2022: 61–83.

[20] Seo H, Brand L, Barco LS, et al. Scaling multi-instance support vector machine to breast cancer detection on the BreaKHis dataset[J]. Bioinformatics, 2022, 38(1): 92–100. 10.1093/bioinformatics/btac267 PMC9235475 35758811

[21] Ak MF. A comparative analysis of breast cancer detection and diagnosis using data visualization and machine learning applications[C]//Healthcare[J]. MDPI, 2020, 8(2): 111–112. 10.3390/healthcare8020111 PMC7349542 32357391

[22] Sharma A, Mishra PK. Performance analysis of machine learning based optimized feature selection approaches for breast cancer diagnosis[J]. International Journal of Information Technology, 2022: 1–12.

[23] Binsaif N. Application of machine learning models to the detection of breast cancer[J]. Mobile Information Systems, 2022: 7340689.

[24] Kourou K, Exarchos TP, Exarchos KP, et al. Machine learning applications in cancer prognosis and prediction[J]. Computational and structural biotechnology journal, 2015, 13: 8–17. 25750696 10.1016/j.csbj.2014.11.005 PMC4348437

[25] Chaabene WB, Flah M, Nehdi ML. Machine learning prediction of mechanical properties of concrete: Critical review[J]. Construction and Building Materials, 2020, 260: 119889.

[26] XIANG YT, WANG M, CAO L. Financial risk prediction method based on multi-objective deep learning model[J]. Statistics&Decision, 2022, 38(10): 184–188.

[27] WAN LY. Educational forecasting in the age of algorithms and the transformation of its research paradigm[J]. Journal of Distance Education, 2022, 40(3): 35–44.

[28] Anisha PR, Reddy CKK, Apoorva K, et al. Early Diagnosis of Breast Cancer Prediction using Random Forest Classifier(C)//IOP Conference Series: Materials Science and Engineering[J]. IOP Publishing, 2021, 1116(1): 012187.

[29] Aroef C, Rivan Y, Rustam Z. Comparing random forest and support vector machines for breast cancer classification[J]. TELKOMNIKA (Telecommunication Computing Electronics and Control), 2020, 18(2): 815–821.

[30] Cocco D, ElSherif A, Wright MD, et al. Invasive lobular breast cancer: data to support surgical decision making[J]. Annals of surgical oncology, 2021, 28: 5723–5729. 34324111 10.1245/s10434-021-10455-7

[31] Chaffer CL, Weinberg RA. A perspective on cancer cell metastasis[J]. science, 2011, 331(6024): 1559–1564. 21436443 10.1126/science.1203543

[32] Ahuja A, Al-Zogbi L, Krieger A. Application of noise-reduction techniques to machine learning algorithms for breast cancer tumor identification[J]. Computers in Biology and Medicine, 2021, 135: 104576. 34246158 10.1016/j.compbiomed.2021.104576

[33] Yu Y, Li M, Liu L, et al. Clinical big data and deep learning: Applications, challenges, and future outlooks[J]. Big Data Mining and Analytics, 2019, 2(4): 288–305.

[34] Yokoyama S, Hamada T, Higashi M, et al. Predicted Prognosis of Patients with Pancreatic Cancer by Machine LearningPrognosis of Pancreatic Cancer by Machine Learning[J]. Clinical cancer research, 2020, 26(10): 2411–2421. 31992588 10.1158/1078-0432.CCR-19-1247

[35] Zhou YY, Chen LP, Zhang Y, et al. Integrated transcriptomic analysis reveals hub genes involved in diagnosis and prognosis of pancreatic cancer[J]. Molecular Medicine, 2019, 25(1): 1–13. 31706267 10.1186/s10020-019-0113-2 PMC6842480

[36] Botsikas D, Bagetakos I, Picarra M, et al. What is the diagnostic performance of 18-FDG-PET/MR compared to PET/CT for the N-and M-staging of breast cancer?[J]. European radiology, 2019, 29: 1787–1798. 30267154 10.1007/s00330-018-5720-8

[37] Liu WC, Li MX, Qian WX, et al. Application of machine learning techniques to predict bone metastasis in patients with prostate cancer[J]. Cancer Manag Res, 2021, 13: 23–36. 10.2147/CMAR.S330591 PMC8627242 34849027

[38] Liu P, Fu B, Yang SX, et al. Optimizing survival analysis of XGBoost for ties to predict disease progression of breast cancer[J]. IEEE Transactions on Biomedical Engineering, 2020, 68(1): 148–160. 32406821 10.1109/TBME.2020.2993278

[39] Prastyo PH, Paramartha IGY, Pakpahan MM, et al. Predicting breast cancer: A comparative analysis of machine learning algorithms[C]. Proceeding International Conference on Science and Engineering, 2020, 3: 455–459.

[40] Liu W, Lee M, Wu SN, et al. Using machine learning methods to predict bone metastases in breast infiltrating ductal carcinoma patients[J]. Frontiers in Public Health, 2022: 2109. 10.3389/fpubh.2022.922510 PMC9298922 35875050

[41] Schettini F, Chic N, Brasó-Maristany F, et al. Clinical, pathological, and PAM50 gene expression features of HER2-low breast cancer[J]. NPJ breast cancer, 2021, 7(1): 1–2. 33397968 10.1038/s41523-020-00208-2 PMC7782714

[42] Zhang H, Katerji H, Turner BM, et al. HER2-low breast cancers: incidence, HER2 staining patterns, clinicopathologic features, MammaPrint and BluePrint genomic profiles[J]. Modern Pathology, 2022, 35(8): 1075–1082. 35184150 10.1038/s41379-022-01019-5

[43] Guven DC, Kaya MB, Fedai B, et al. HER2-low breast cancer could be associated with an increased risk of brain metastasis[J]. International Journal of Clinical Oncology, 2021: 1–8. 10.1007/s10147-021-02049-w 34661778

[44] Qi X, Du J, Tang P, et al. Clinical significance of internal mammary lymph node metastasis for breast cancer: analysis of 337 breast cancer patients[J]. Surgical Oncology, 2018, 27(2): 185–191. 29937170 10.1016/j.suronc.2018.03.006

[45] Yazdani A, Dorri S, Atashi A, et al. Bone metastasis prognostic factors in breast cancer[J]. Breast Cancer, 2019, 13:1178223419830978. 10.1177/1178223419830978 PMC6388452 30828246

[46] Poortmans PM, Collette S, Kirkove C, et al. Internal mammary and medial supraclavicular irradiation in breast cancer[J]. New England Journal of Medicine, 2015, 373(4): 317–327. 26200978 10.1056/NEJMoa1415369

[47] Lee SE, Yoon JH, Son NH, et al. Screening in Patients With Dense Breasts: Comparison of Mammography, Artificial Intelligence, and Supplementary Ultrasound[J]. American Journal of Roentgenology, 2023: 1–11. 37493324 10.2214/AJR.23.29655

