# Supplementary material for: Machine learning-based prediction of distant metastasis risk in invasive ductal carcinoma of the breast
Source: PLoS One. 2025 Feb 26;20(2):e0310410. doi: 10.1371/journal.pone.0310410 (PMC11864521; doi:10.1371/journal.pone.0310410)
Supplement: Attachment — Submitted filename: PONE-D-23-20544 Reviewer.pdf [file pone.0310410.s002.pdf]

Reviewer Comments

Journal: PLOS ONE
Manuscript Title: Machine learning-based prediction of distance metastasis risk in invasive ductal carcinoma of the breast
Manuscript ID: PONE-D-23-20544
Submission Date: Sunday, July 30, 2023

The manuscript presented "a prediction approach of distance metastasis risk in invasive ductal carcinoma of the breast". However, the major and critical weak points are that:

(1) Their proposed work discussion is weak distributed to be described or analyzed.
(2) The novelty is not guaranteed.
(3) Their experiments leak from the descriptive and statistical analysis.

The rest of my review presents other weak points, comments, and opinions in detail.

Overall Comments:

(1) [**KEYWORDS**] The keywords (i.e., index terms) should be sorted in alphabetical order.
(2) [**ABSTRACT**] The abstract should contain the best-achieved results from the performed experiments.
(3) [**ABSTRACT**] The abstract should reflect the contributions of the manuscript. I suggest rewriting it.
(4) [**INTRODUCTION**] The authors should provide a clear problem definition and contributions in the introduction section.
(5) [**RESEARCH QUESTION**] Where is the research question and research gap?
(6) [**RESEARCH QUESTION**] The research question is not well-formulated or is poorly motivated, and the paper does not provide new insights or information that is not already known.
(7) [**RELATED WORK**] Where are the related studies? They should be declared in a separate section.
(8) [**RELATED WORK**] A table of comparisons should be added at the end of the related studies section to praise the pros. and cons. of them. The year column should be added and they should be ordered by it.
(9) [**EQUATIONS**] The authors should follow the journal authors' guidance in writing the equations, symbols, and variables. Please, refer to the authors guidelines on the journal official website.
(10) [**EQUATIONS**] Where are the equations of the used metrics?
(11) [**DATASETS**] Samples from the used dataset should be added and annotated.
(12) [**METHODOLOGY**] The suggested approach is not clearly discussed. More scientific details should be added.
(13) [**METHODOLOGY**] What are the used equations in the suggested approach? In other words, how the suggested approach is derived?
(14) [**METHODOLOGY**] Where is the overall pseudocode? Flowchart? of the suggested approach?

(15) [**EXPERIMENTS**] The working environment (i.e., software and hardware) should be declared and added to a table.

(16) [**EXPERIMENTS**] The experimental configurations (i.e., settings) should be declared and added to a table.

(17) [**EXPERIMENTS**] What are the criteria for selecting the experimental configurations?

(18) [**EXPERIMENTS**] More experiments should be conducted using different configurations.

(19) [**EXPERIMENTS**] Where is the tabular representation of the reported results?

(20) [**EXPERIMENTS**] Why did not the authors compare their approach with others in a table?

(21) [**EXPERIMENTS**] Why did not the authors compare their approach with another approach to compare the suggested approach efficiency and applicability?

(22) [**EXPERIMENTS**] Where is the detailed and statistical discussion of the reported results?

(23) [**EXPERIMENTS**] More experiments should be conducted using a different dataset to prove the generalization.

(24) The manuscript does not provide a clear contribution to the field of research.

(25) The manuscript does not provide a clear justification for the research.

(26) [**RESULTS**] The authors have not provided adequate visual aids, such as graphs or tables, to help readers understand the data.

(27) [**ABBREVIATIONS**] The authors should add a table of abbreviations in the revised manuscript.

(28) [**SYMBOLS**] The authors should add a table of symbols in the revised manuscript.

(29) [**CONCLUSIONS**] The conclusions in this manuscript are primitive. Please, write your conclusions.

(30) [**REFERENCES**] There are no citations for many sentences in the manuscript. Why? Please check.

(31) [**REFERENCES**] The references should be written in the same style following the journal authors' guidance.

(32) [**REFERENCES**] Recent citations from 2021 and 2022 should be added to the manuscript.

(33) [**PROOFING**] The authors should get editing help from someone with full professional proficiency in English.

(34) [**PROOFING**] The manuscript should be checked again to fix any typos such as missing spaces and commas.

(35) [**CONSISTENCY**] The manuscript structure is too short. It must be elaborated in their applied technology as should support more rigorous technical aspects.

(36) [**CONSISTENCY**] Some paragraphs are wrapped in more than 10 lines. They should be split concisely.

(37) [**NOVELTY**] What is the novelty of the suggested approach?

(38) [**LIMITATIONS**] What are the limitations of the current study? It should be added in a separate section.

Reviewer Comments

For the authors in case of the authors got a chance to review the manuscript and submit the revised one after the editor's decision, please, provide a table in the revised manuscript mentioning (1) the comment, (2) the authors' response, and (3) the authors' change (if applicable). Please, consider all of the comments and don't ignore any of them.

*Please, refer to the attached file "**PONE-D-23-20544 Reviewer.pdf**" for the same comments in an organized format.*
