# Supplementary material for: Machine learning-based prediction of distant metastasis risk in invasive ductal carcinoma of the breast
Source: PLoS One. 2025 Feb 26;20(2):e0310410. doi: 10.1371/journal.pone.0310410 (PMC11864521; doi:10.1371/journal.pone.0310410)
Supplement: Attachment — Submitted filename: PONE-D-23-20544_reviewer (3).pdf [file pone.0310410.s003.pdf]

PLOS ONE

Machine learning-based prediction of distance metastasis risk in invasive ductal carcinoma of the breast

--Manuscript Draft--

**Manuscript Number:**	PONE-D-23-20544
**Article Type:**	Research Article
**Full Title:**	Machine learning-based prediction of distance metastasis risk in invasive ductal carcinoma of the breast
**Short Title:**	Machine learning-based prediction of distance metastasis risk in invasive ductal carcinoma of the breast
**Corresponding Author:**	li Zhang<br>Shihezi University<br>Shihezi, CHINA
**Keywords:**	machine learning; Infiltrating ductal carcinoma; cancerometastasis; prediction; data mining
**Abstract:**	Invasive ductal carcinoma (IDC) of the breast is the most common pathological type of breast cancer, which is prone to distant organ metastasis. Clinically, more than 90% of the deaths caused by breast cancer are due to metastasis related complications. Therefore, early identification and diagnosis of whether IDC has distant metastasis is extremely important. The study aimed to use machine learning technology to establish an effective prediction model to predict the the risk of distant organ metastasis in IDC, which can help clinical doctors make fast and reliable diagnoses, accurately identify high-risk patients, enable patients to receive treatment in a timely manner, and improve survival opportunities. This study is based on four algorithms, namely random forest, XGBoost, logical regression, and support vector machine (SVM) to construct risk prediction models, and used these four machine learning models as the basic model to build a fusion model based on the voting mechanism. Using accuracy, precision, recall, F1-score, etc. as indicators to evaluate model performance. Results proved that the IDC remote transfer risk prediction fusion model has has good performance. This stable risk prediction model provides a meaningful reference support for doctors in identifying and diagnosing the risk of IDC hematogenous metastasis, while improving the work efficiency of doctors and striving for more survival opportunities for patients.
**Order of Authors:**	Jingru Dong
	Ruijiao Lei
	Feiyang Ma
	Lu Yu
	Lanlan Wang
	Shangzhi Xu
	Yunhua Hu
	Jialin Sun
	Wenwen Zhang
	Haixia Wang
	li Zhang
**Additional Information:**	
**Question**	**Response**
**Financial Disclosure**<br><br>Enter a financial disclosure statement that	The author(s) received no specific funding for this work.

Powered by Editorial Manager® and ProduXion Manager® from Aries Systems Corporation

describes the sources of funding for the work included in this submission. Review the submission guidelines for detailed requirements. View published research articles from *PLOS ONE* for specific examples.<br><br>This statement is required for submission and **will appear in the published article** if the submission is accepted. Please make sure it is accurate.<br><br>**Unfunded studies**<br>Enter: *The author(s) received no specific funding for this work.*<br><br>**Funded studies**<br>Enter a statement with the following details:<br>• Initials of the authors who received each award<br>• Grant numbers awarded to each author<br>• The full name of each funder<br>• URL of each funder website<br>• Did the sponsors or funders play any role in the study design, data collection and analysis, decision to publish, or preparation of the manuscript?<br>• **NO** - Include this sentence at the end of your statement: *The funders had no role in study design, data collection and analysis, decision to publish, or preparation of the manuscript.*<br>• **YES** - Specify the role(s) played.<br><br>* typeset	
**Competing Interests**<br><br>Use the instructions below to enter a competing interest statement for this submission. On behalf of all authors, disclose any competing interests that could be perceived to bias this work—acknowledging all financial support and any other relevant financial or non-financial competing interests.<br><br>This statement is required for submission and **will appear in the published article** if the submission is accepted. Please make sure it is accurate and that any funding	The authors have declared that no competing interests exist.

sources listed in your Funding Information later in the submission form are also declared in your Financial Disclosure statement.<br><br>View published research articles from *PLOS ONE* for specific examples.<br><br>**NO authors have competing interests**<br><br>Enter: *The authors have declared that no competing interests exist.*<br><br>**Authors with competing interests**<br><br>Enter competing interest details beginning with this statement:<br><br>*I have read the journal's policy and the authors of this manuscript have the following competing interests: [insert competing interests here]*<br><br>* typeset	
**Ethics Statement**<br><br>Enter an ethics statement for this submission. This statement is required if the study involved:<br><br>• Human participants<br>• Human specimens or tissue<br>• Vertebrate animals or cephalopods<br>• Vertebrate embryos or tissues<br>• Field research<br><br>Write "N/A" if the submission does not require an ethics statement.<br><br>General guidance is provided below. Consult the submission guidelines for detailed instructions. **Make sure that all information entered here is included in the Methods section of the manuscript.**	N/A

**Format for specific study types**<br><br>**Human Subject Research (involving human participants and/or tissue)**<br>• Give the name of the institutional review board or ethics committee that approved the study<br>• Include the approval number and/or a statement indicating approval of this research<br>• Indicate the form of consent obtained (written/oral) or the reason that consent was not obtained (e.g. the data were analyzed anonymously)<br><br>**Animal Research (involving vertebrate animals, embryos or tissues)**<br>• Provide the name of the Institutional Animal Care and Use Committee (IACUC) or other relevant ethics board that reviewed the study protocol, and indicate whether they approved this research or granted a formal waiver of ethical approval<br>• Include an approval number if one was obtained<br>• If the study involved *non-human primates*, add *additional details* about animal welfare and steps taken to ameliorate suffering<br>• If anesthesia, euthanasia, or any kind of animal sacrifice is part of the study, include briefly which substances and/or methods were applied<br><br>**Field Research**<br><br>Include the following details if this study involves the collection of plant, animal, or other materials from a natural setting:<br>• Field permit number<br>• Name of the institution or relevant body that granted permission	
**Data Availability**<br><br>Authors are required to make all data underlying the findings described fully available, without restriction, and from the time of publication. PLOS allows rare exceptions to address legal and ethical concerns. See the PLOS Data Policy and FAQ for detailed information.	Yes - all data are fully available without restriction

A Data Availability Statement describing where the data can be found is required at submission. Your answers to this question constitute the Data Availability Statement and **will be published in the article**, if accepted.<br><br>**Important:** Stating 'data available on request from the author' is not sufficient. If your data are only available upon request, select 'No' for the first question and explain your exceptional situation in the text box.<br><br>Do the authors confirm that all data underlying the findings described in their manuscript are fully available without restriction?	
**Describe where the data may be found in full sentences. If you are copying our sample text, replace any instances of XXX with the appropriate details.**<br><br>• If the data are **held or will be held in a public repository**, include URLs, accession numbers or DOIs. If this information will only be available after acceptance, indicate this by ticking the box below. For example: *All XXX files are available from the XXX database (accession number(s) XXX, XXX.).*<br>• If the data are all contained **within the manuscript and/or Supporting Information files**, enter the following: *All relevant data are within the manuscript and its Supporting Information files.*<br>• If neither of these applies but you are able to provide **details of access elsewhere**, with or without limitations, please do so. For example:<br><br>*Data cannot be shared publicly because of [XXX]. Data are available from the XXX Institutional Data Access / Ethics Committee (contact via XXX) for researchers who meet the criteria for access to confidential data.*<br><br>*The data underlying the results presented in the study are available from (include the name of the third party*	All relevant data are within the Supporting Information files. Raw data from SEER database.

and contact information or URL).<br>• This text is appropriate if the data are owned by a third party and authors do not have permission to share the data.<br><br>* typeset	
Additional data availability information:	

Powered by Editorial Manager® and ProduXion Manager® from Aries Systems Corporation

Manuscript Click here to access/download;Manuscript;manuscript.docx

Machine learning-based prediction of distant metastasis risk in invasive ductal carcinoma of the breast

Jingru Dong[1], Ruijiao Lei[1], Feiyang Ma[1], Lu Yu[2], Lanlan Wang[2], Shangzhi Xu[2], Yunhua Hu[2], Jialin Sun[2], Wenwen Zhang[2], Haixia Wang[2] and Li Zhang[2]*

*Corresponding Author: Li Zhang
[1,2,*]Shihezi University Medical College School of Medical College, Shihezi University, Xinjiang, China
Phone number: 13239934563
Fax number: 13239934563
Email address: zl491191385@163.com

Abstract

Invasive ductal carcinoma (IDC) of the breast is the most common pathological type of breast cancer, which is prone to distant organ metastasis. Clinically, more than 90% of the deaths caused by breast cancer are due to metastasis related complications. Therefore, early identification and diagnosis of whether IDC has distant metastasis is extremely important. The study aimed to use machine learning technology to establish an effective prediction model to predict the the risk of distant organ metastasis in IDC, which can help clinical doctors make fast and reliable diagnoses, accurately identify high-risk patients, enable patients to receive treatment in a timely manner, and improve survival opportunities. This study is based on four algorithms, namely random forest, XGBoost, logical regression, and support vector machine (SVM) to construct risk prediction models, and used these four machine learning models as the basic model to build a fusion model based on the voting mechanism. Using accuracy, precision, recall, F1-score, etc. as indicators to evaluate model performance. Results proved that the IDC remote transfer risk prediction fusion model has has good performance. This stable risk prediction model provides a meaningful reference support for doctors in identifying and diagnosing the risk of IDC hematogenous metastasis, while improving the work efficiency of doctors and striving for more survival opportunities for patients.

Key words: machine learning; Infiltrating ductal carcinoma; cancerometastasis; prediction; data mining

1. Introduction

Breast cancer (BC) is the most common malignant tumor and the main cause of

cancer-related deaths in women worldwide [1]. According to data from the International Agency for Research on Cancer (IARC) of the World Health Organization, 19.29 million people will be newly diagnosed with cancer in the world in 2020, of which 2.26 million will be newly diagnosed with BC, ranking this first among the tumors. A recent epidemiological study predicts that the global incidence rate of breast cancer will reach about 3 million cases per year by 2050 [2]. Among the new cases of female malignant tumors, breast cancer accounts for as high as 30%, and the mortality rate ranks second in cancer mortality [3,4]. In China, breast cancer is the malignant tumor with the highest morbidity and mortality among Chinese women. There are about 420000 new cases every year. The overall 5-year survival rate of patients with advanced breast cancer is less than 50%. Infiltrating Ductal Carcinoma (IDC) is the most common subtype among breast cancer, accounting for about 80% of all breast cancer cases [5,6], and is also the most common form of invasive breast cancer. This histopathological type of breast cancer has a poor prognosis, is highly prone to axillary lymph node metastasis and distant organ metastasis, and the five-year survival rate of patients with distant metastasis of invasive ductal carcinoma is only 27.4%. However, clinically, many patients are diagnosed in the middle and advanced stages, which are prone to local recurrence and distant organ metastasis, and even if the treatment is strengthened, the efficacy is far less than the previous stage. Terefore, early detection of breast cancer with distant metastasis can develop individualized treatment plans, which have specific significance for predicting the survival time of patients and improving the prognosis of

patients [7]. Risk assessment of cancer metastasis is important to reduce overall breast cancer mortality. Appropriate risk assessment is a major factor in the management of breast cancer that determines a patient's viability [8].

The successful introduction of information and communication technologies (ICT) in medical practice is an important stake in the renovation of the health system and more precisely in cancer care [9]. With the development of artificial intelligence, machine learning has shown a very strong data mining ability and has been applied to all walks of life. In recent years, machine learning algorithms have been widely used in face recognition, industrial prediction and other applications. Combining machine learning with medical problems and integrating this development, more intelligent auxiliary diagnosis methods can be proposed to increase treatment time and cure opportunities for patients. In recent years, machine learning has proven to be of great value in BC diagnosis and survival, prognosis prediction, and other aspects [10,11]. The use of machine learning algorithms to predict the survival risk of BC is currently a research hotspot at the intersection of artificial intelligence and medicine [12,13]. However, few studies have focused on combining machine learning to predict the risk of distant metastasis of cancer cells in IDC. In this study, a machine learning algorithm was used to replace the traditional statistical analysis method to process the clinical characteristic data of IDC patients, and a prediction model was developed to discover or explain the relationship between some independent variables and whether patients with invasive ductal carcinoma of the breast are at risk of developing distant organ

metastases. This study focused on four models, namely logistic regression, SVM, random forest, and XGBoost, and integrated these four machine learning models as base models to construct hybrid models, and then used cross-validation technology to compare the accuracy, recall, and AUC value of the model prediction. The highly effective predictive model is useful for breast surgeons to quickly and accurately determine whether distant metastases of cancer cells have occurred in IDC patients.

2. Materials and Methods

2.1 Construction of the Dataset

2.1.1 Data Acquisition

The data used in this experiment were obtained using SEER * stat software (version8.4.0.1, National Cancer Institute, American) based on the surveillance, epidemiology, and final results (SEER) database, using the "International Classification of Oncological Diseases, Third Edition (ICD-O-3)." The inclusion criteria were as follows: (1) tumor location: site recode ICD-O-3 is broken; (2) the pathological tissue ICD-O-3 classification was 8500/3 (IDC); (3) The 8th edition of the American Joint Committee of Cancer (AJCC) TNM staging criteria was used for tumor staging; and (4) Complete clinical, pathological and follow-up data are available. The exclusion criteria were as follows: (1) tumor location: site recode ICD-O-3 is broken; (2) the pathological tissue ICD-O-3 classification was 8500/3 (IDC); and (3) female patients. The exclusion criteria were as follows: (1) missing or unknown disease-related information; (2) age <20 or >85 years; (3) T, N, and M stage, race, grade, marital status, chemotherapy status,

radiotherapy status, and other unknown information; and (4) incomplete follow-up information.

2.1.2 Data Preprocessed

Data mining and analysis, were conducted using Python (version 3.10, Python Software Foundation, Netherlands). For data preprocessing: (1) Read data set: After screening the data set, there were no missing, abnormal, duplicate, or other phenomena of the data. (2) Introduced the function RandomUndersample () which is used for random undersampling from the imblearn unbalanced-learn library, and used this undersampling method to resample the data. (3) The classification variables should be dummy coded and converted into virtual variables. (4) Training in scikit-learn_ test_ Split () randomly divides the data into training sets and test sets at a ratio of 8:2. (5) StandardScaler is used to standardize the transformed dataset to eliminate the dimensional relationship between variables and make the data comparable. (6) SelectFromModel() in the feature_selection module, based on the Pearson correlation coefficient principle, calculates the correlation coefficient between two features, and selects a small number of features to represent the characteristics of the data, so as to enhance the generalization ability of the classifier and avoid overfitting the data. Effective data preprocessing is a prerequisite for establishing and training the following model.

2.2 Model Establishment

2.2.1 Introduction to Algorithm Principle

Support vector machines (SVMs) are supervised learning algorithms that can deal

very successfully with many problems such as regression problems and pattern recognition [14]. The SVM is essentially a two-classification classifier [15]. Its basic model is a linear classifier with the largest interval defined in the feature space. The basic idea is to solve a separate hyperplane that can correctly divide the training dataset which has the largest geometric interval, and uses the hyperplane to distinguish different groups. This is a powerful machine learning technique based on 3D and 2D modeling [16,17].

Logistic Regression (LR) has made great progress in many fields, especially in the medical field. By establishing a functional relationship between two or more predictive variables and one outcome variable. The expression of this function is given by the formula (1), which uses the regression model to predict the probability that a given data object or entry belong to given category [18]. The closer f (z) is to 1, the greater the probability that the result is 1, and that the probability value is equal to f (z). To solve the classification problem, logical regression needs to find a sufficiently differentiated decision boundary, determine the relationship between the classification probability and the input variables on this basis, and then determine the category to which it belongs by comparing the probability value.

$$f(z) = \frac{1}{1+e^{-2}} \tag{1}$$

Random forest (RF) is a bagging ensemble learning algorithm. It uses randomly selected training samples and variable subsets to generate multiple decision trees. Each decision tree is a classifier that produces the classification results. The basic idea is to

randomly select "n" subsamples from the total samples of the training set, establish "n" decision trees for "n" subsamples according to the one-to-one mapping relationship, vote the optimal results of each tree, and then perform the final classification of the test object. By forming a lot of tree into random forest the risk of getting over fitting is reduced, with the error and converge into some value generalized [19,20,21]. as shown in Fig 1.

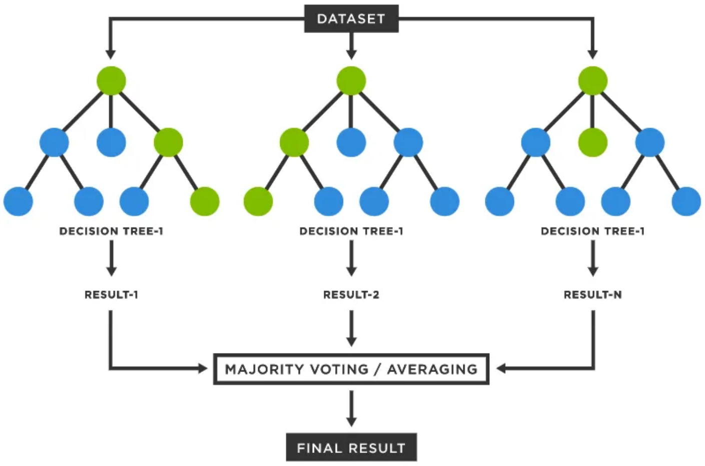

Fig 1. Schematic diagram of Random forest

EXtreme Gradient Boosting (XGB) is a boosting integrated learning algorithm that is a gradient lifting algorithm based on the CART regression tree. After multiple iterations, each iteration produces a weak classifier, and each classifier is trained based on the gradient of the previous classifier round. The basic idea can be understood as taking the second-order Taylor expansion of the loss function as its replacement function. The objective function of the algorithm is composed of the loss function L of the model and the regular term Ω to restrain the complexity of the model.

The loss function can be expressed by the predicted and real values, as shown in

Equation (2):

$$L = \sum_{i=1}^{n} l(y_{i,}\widehat{y_i}) \tag{2}$$

Where: n is the number of samples.

The objective function is formula (3):

$$Obj = \sum_{k=1}^{k} f_k(x_i) + \sum_{i=1}^{k} \Omega(f_i) \tag{3}$$

Where Ω is the regular term of the model.

Finally, this study also built a fusion model of the four algorithms mentioned above based on a voting mechanism, and the final output is theoretically the most popular prediction among the base learners. The basic principle is shown in Figure 2, that is, the average of the probability of all model prediction samples for a certain category is used as the criterion, and the corresponding type with the highest probability is the final prediction result.

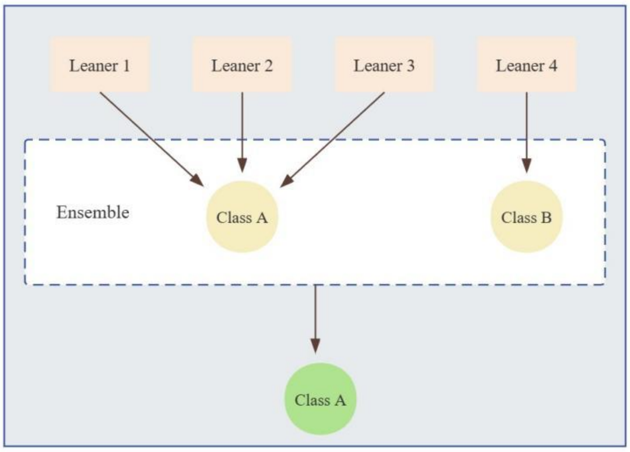

Fig 2. Schematic diagram for establishing a fusion model based on voting mechanism

2.2.2 Model Establishment Process

The training and testing procedures of the machine learning models were

demonstrated in the JupyterNotebook environment using Python. Python sklearn and pandas software packages were used for data analysis, and Python matplotlib and seaborne were used to draw graphics. The basic modelling process is illustrated in Figure 3.

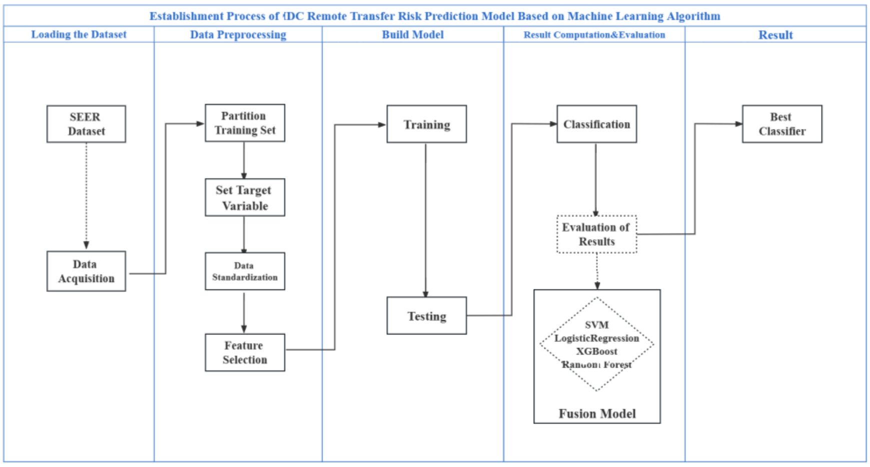

Fig 3. Modeling flowchart

3. Result

3.1 Evaluation Indicators

A confusion matrix is used to reflect the correct and wrong classification of patients by each classifier. Specific evaluation indicators include accuracy, recall, precision, F1-score, AUC and learning curve to evaluate the performance of the model. The calculation formula for each indicator is shown in the formulas (4)-(8).

$$Accuracy = \frac{TP+TN}{TP+FN+FP+TN} \tag{4}$$

$$Precision = \frac{TP}{TP+FP} \tag{5}$$

$$Recall = \frac{TP}{TP+FN} \tag{6}$$

$$F1 - score = \frac{2*Precision*Recall}{Precision+Recall} \tag{7}$$

$$AUC = \frac{\sum R_i(I_p) - I_p(I_p+1)/2}{I_p + I_n} \quad (8)$$

TP refers to true positives, TN refers to true negatives, FP refers to false positives, FN refers to false negatives, and the Fl value is an indicator used to measure the accuracy of the binary classification model in statistics. This score takes account both of the accuracy rate and recall rate of the model. AUC：The area under the curve is a numerical value that tells us how will the model perform in different situations. The state of the model can be intuitively understood by observing the drawn learning curve. For example, overfitting or underfitting can occur.

3.2 Demographic Characteristics Analysis

Data Visualization is an extremely necessary skill in machine learning. It is responsible for providing a qualitative understanding of the given data. Figure 4 shows the distributions of the four continuous variables, it could be seen that 65-70 years old is the high incidence of cancer metastasis in IDC patients. Meanwhile, patients with hematogenous metastases have more lymph node detections and positive local lymph nodes. Tumor size is concentrated between 0-100mm.

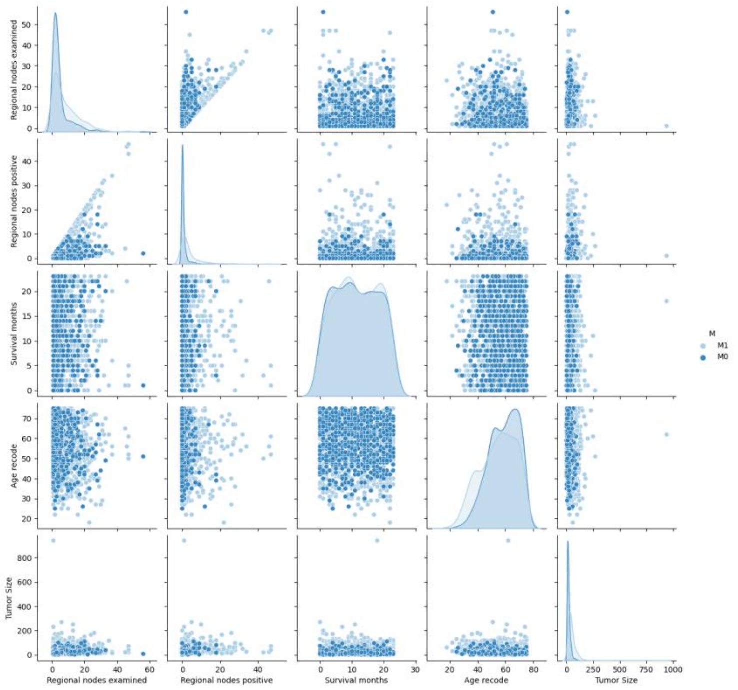

Fig 4. Continuous variable distribution map

3.3 Correlation Analysis of Features

To determine the impact of these features on the prediction, a feature correlation test was conducted. Correlation analysis between the characteristic variables of the dataset shows the degree of interaction between the variables. This study uses a thermal matrix diagram to show the correlation between these features and the prediction of the machine learning (ML) algorithm. Figure 5 shows the correlation matrix of 14 variables on the training set after dimensionality reduction. The matrix displays the correlation between variables.Each cell in the matrix shows the Spearman correlation coefficient between two variables. From this figure, the Tumor size, T stage, Radiation and Chemotherapy records, Regional nodes positive, and other characteristic variables were highly correlated. This is consistent with the clinical experience, indicating that it is

more invasive.

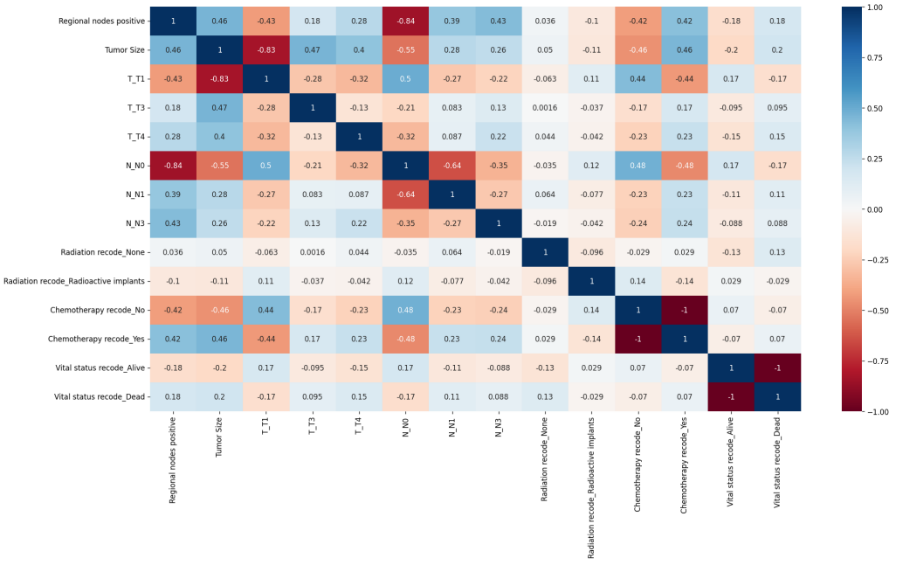

Fig 5. Characteristic variable correlation thermal matrix diagram

3.4 Model Performance

Figure 6 shows the classification of the data by each classifier. Through comparison, we can see that the fusion prediction model has the least error classification. The prediction performance of all models was compared by 10-fold cross validation of the accuracy, precision, recall and F1 value on the training set. The results are shown in Figure 7-8. In a comprehensive view, all indicators of the fusion model are high, and its accuracy, Recall, Precision, and F1 mean values are 83.5%, 83.8%, 85.8%, and 83.9%, respectively. from Figure 9, we can see that the AUC value of the fusion model even reaches 96%. The evaluation of the model state using the learning curve is shown in Figure 10, and after cross-validation, we obtain the same result: the fusion model fits converge relatively best.

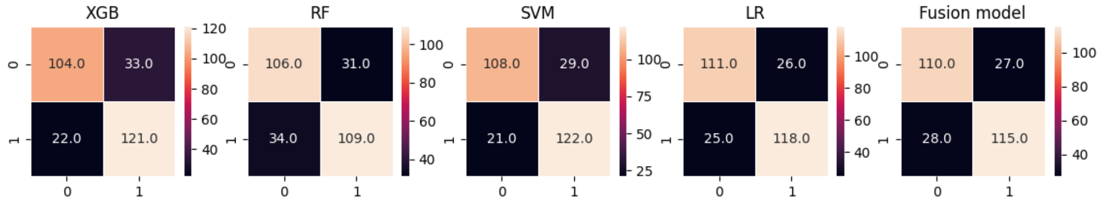

Fig 6. Confusion matrix diagram of each classifier

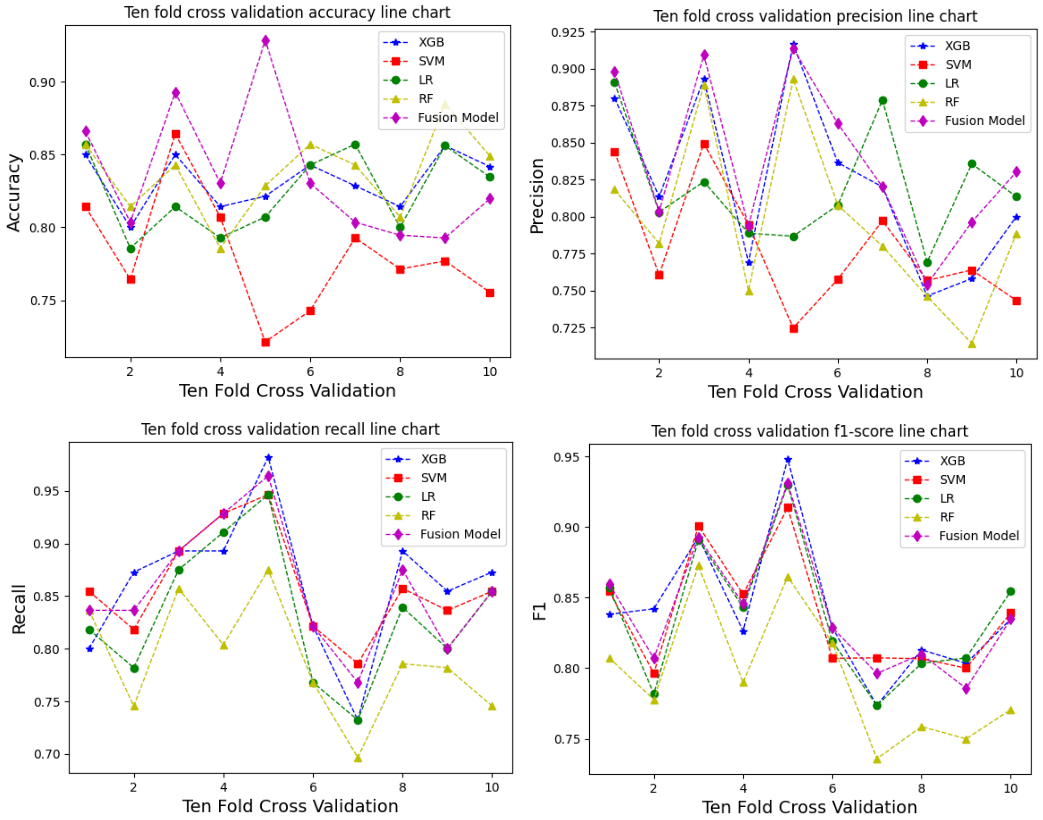

Fig 7. Line chart of ten fold cross validation results of each prediction model

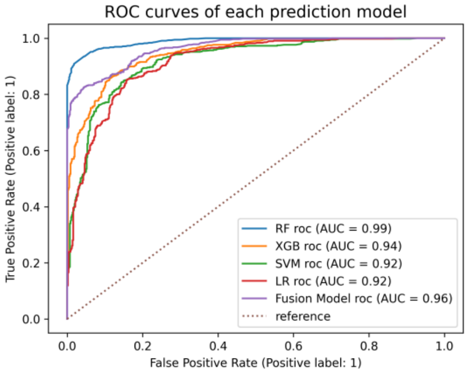

Fig 8. Receiver operating characteristic of each prediction model

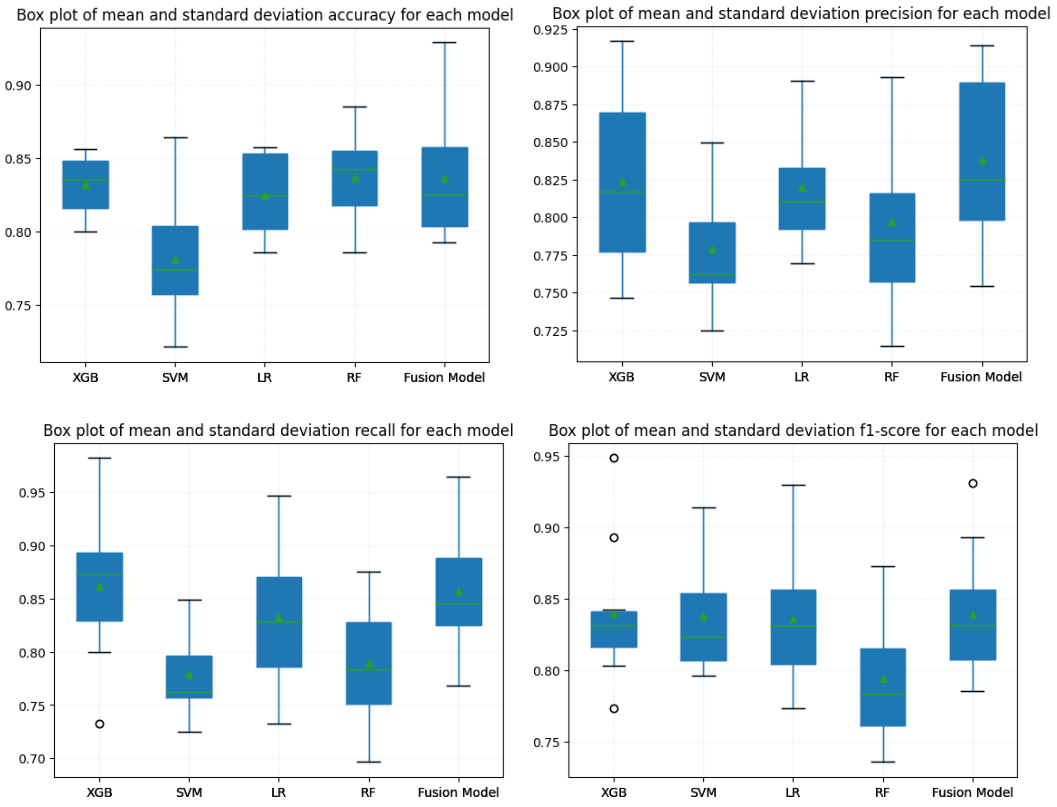

Fig 8. Box plot of mean and standard deviation for each indicator

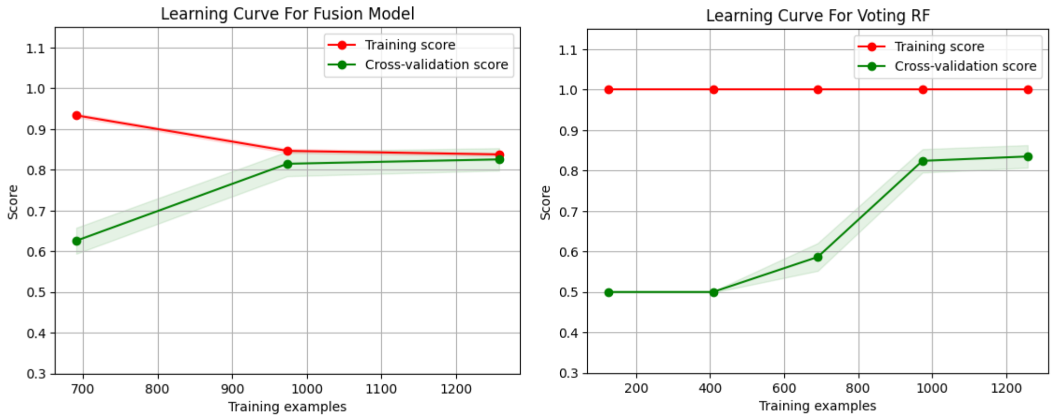

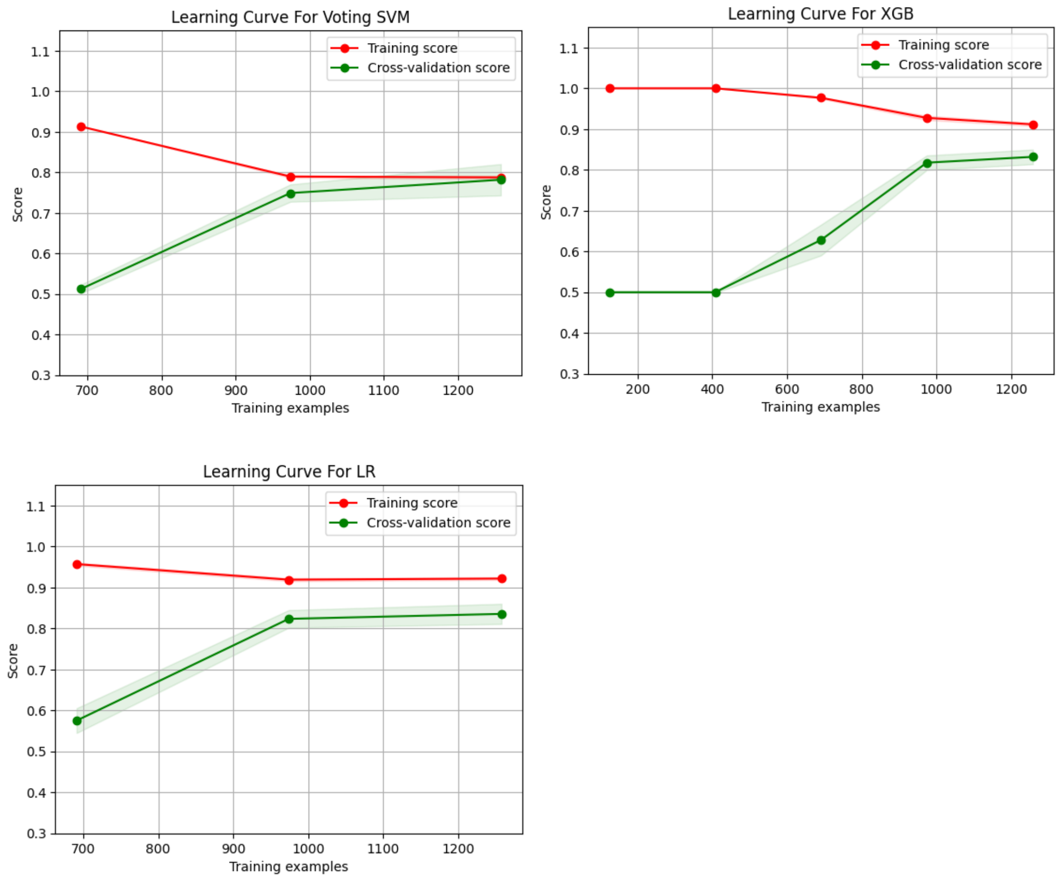

Fig 9. Learning curve of each prediction model

3.6 Importance: Analysis of Characteristic Variables

Based on the five prediction models, this study ranked the importance of 14 feature variables after dimensionality reduction processing on the training set, as shown in Figure 11. Overall, the weight of feature variables such as N, T-stage, tumor size, and radiotherapy records was much higher than other variables.

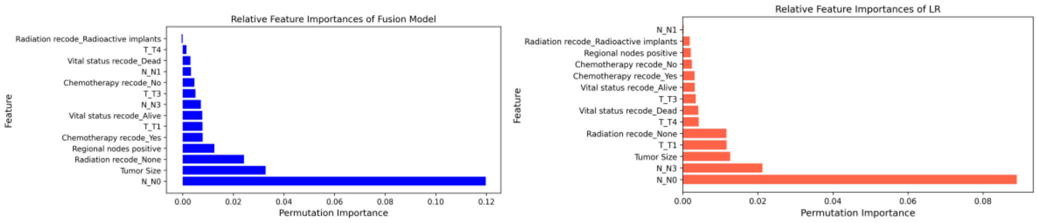

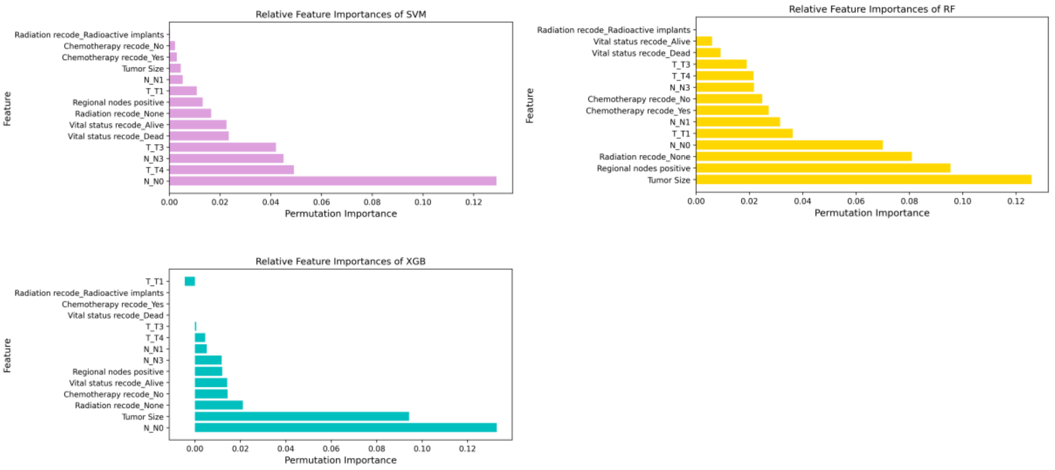

Fig 10. Contributions of the clinical features variables to risk prediction using Feature importance.

4. Discussion

Over the past few decades, the incidence of death in breast cancer patients has decreased significantly due to advances in surgical methods and systematic neoadjuvant chemotherapy [22]. However, distant organ metastases remain the biggest obstacle to treatment for breast cancer. In clinical practice, when hematogenous metastases are detected, it indicates that the patient is already at an advanced stage and the five-year survival rate may drop by nearly 30% due to late diagnosis [23]. Therefore, early identification of patients with IDC at risk of developing distant metastases from cancer is extremely important. In the actual treatment process, pathological examination is the gold standard for metastatic tumors. When a physician suspects that a patient has metastatic cancer somewhere, he or she will usually first have the patient undergo a series of immunohistochemical tests to aid in the diagnosis; not only will this invasive test be more physically harmful to the patient, but the pathology results are usually

available two to three days after the test, and this waiting time is critical to improving the survival chances of patients with advanced hematogenous metastatic breast cancer [24]. This study uses only clinical and pathological indicators of IDC patients obtained based on the SEER database to establish a validated prediction model to classify and predict whether a patient has cancer metastasis, which can easily predict the risk of organ metastasis without imaging or any laboratory tests, which can help clinicians make a rapid and reliable diagnosis and improve their work efficiency, and can develop individualized treatment plans for different individuals [25], and save treatment time for patients by identifying the risk of metastasis early, thus reducing mortality.

Four machine learning models, logistic regression, SVM, random forest and XGBoost, were selected in this study, and the results after ten-fold cross-validation showed that the accuracy: FM>RF>XGB>LR>SVM; precision: FM>XGB>LR>RF>SVM; recall: XGB>FM>SVM>LR>RF; F1 value: FM≈XGB≈LR≈SVM>RF; AUC: RF>FM>XGB>SVM>LR. Overall, each prediction model has its own strengths and weaknesses. The random forest model has a high prediction accuracy and AUC, which indicates a high overall prediction accuracy, while this classifier has a high ability to rank the samples, but the precision, recall, and F1 values of this model are low, which indicates its low prediction accuracy in positive sample results, while there are cases of missing diagnoses. For the LR and SVM models, all metrics are low and unsuitable as a good classifier for risk prediction. The performance of the fusion model based on the voting mechanism was improved to a certain extent in

all indicators compared to each base model. The high accuracy indicates that the model can classify patients more accurately, the high sensitivity implies a low rate of missed diagnoses, and the small error indicates the stable predictive performance of the model. Therefore, the Fusion classifier is more suitable as a good predictive model to predict the risk of distant organ metastasis in IDC.

This study used an under-sampling method to solve the problem of data imbalance during the data preprocessing, which can make the prediction model more balanced to a certain extent, but will also lose a lot of data information, which can easily cause over-fitting of the model. Therefore, this experiment used a learning curve to diagnose the overfitting behavior of the model. As shown in Figure 12, with an increase in the number of training samples, the accuracy difference between LR, XGB and RF on the training and cross-validation sets is large, indicating that the models can fit the known data well, but the generalization ability is poor and they are over-fitted. The curves on both the FM and SVM training and validation sets were able to converge, but the SVM was only 0.75, with a slightly larger deviation, and the fit was not satisfactory. At this time, the FM convergence value is 0.82, which indicates that the algorithm itself has good fitting ability, the model has strong generalization ability, and the fitting effect valence is ideal. Consistent with the conclusions drawn earlier, the fusion prediction model based on the voting mechanism not only has superior performance, but also has strong model fitting and generalization capabilities.

Figure 13 illustrated the importance ranking of the characteristic variables for the

five ML models used to predict IDC distant transfer risk. The ranked importance principle is used to analyze the relative characteristic importance of the variables in each ML model [26]. We found that although the relative feature importance varied slightly in different ML models, T, N stage (especially N0, T3, and T4 stages), and Tumor Size were the top-ranked variables in most models, compared to the Rediation recode variable, which was last in most models, but it also contributed to the models. Through the importance analysis of the characteristic variables, we know that five variables, T and N staging, Tumos Size, Regional nodes positive, and Radiation recode, are strong predictors of IDC distant transfer risk and have good predictive value. It can also suggest which variables are critical for the creation of the predictive model in this experiment. These characteristic variables can be considered for inclusion in future studies when conducting such experiments to provide a reference when selecting variables for the database.

Although some BC prediction models based on the SEER database have been developed previously, few studies have attempted to use the ML algorithm to predict the risk of distant IDC transfers. Therefore, in this study, we built a model with good predictive performance and good ability to fit the data for predicting the risk of cancer metastasis in IDC patients, based on data from the SEER database and incorporating four machine learning models. Our predictive model allows clinicians to easily input relevant clinical and pathological indicators of their patients into the model, which will calculate the patient's risk of cancer metastasis and then make quick and accurate

personalized decisions for their patients based on the predictive output, and can be used as the basis for clinicians to explain their decisions to patients and engage them in their treatment choices.

5. Conclusion

The goal of this study is to develop a risk prediction model that can predict the future risk of cancer metastasis in patients with IDC using only the available data information. we used real IDC breast cancer dataset samples to construct a fusion model based on SVM, logistic regression, random forest, and XGBoost four classical machine learning based on voting mechanism. This risk prediction model has superior predictive performance and high medical application value, which can be used as a better tool to assist in assessing the risk of distant metastasis of IDC, helping clinicians to improve efficiency and reduce the misdiagnosis rate, while maximizing the risk of death from IDC by buying more treatment time for patients with advanced stages. However, the data used in this study were limited by the region, quantity, and subjectivity of the feature selection, which limited the final experimental results. We can consider further expanding the data source and using large sample data or selecting feature variables more accurately when screening datasets to verify the applicability of the results. Moreover, this was a retrospective study that needs to be verified through prospective research.

Acknowledgements

The authors would like to express deep sense of appreciation and heartfelt thanks

the public database provided by the National Cancer Institute, American, for providing data support for this study. We would like to thank Editage (www.editage.cn) for English language editing.

Author Contributions

Jingru Dong: Data acquisition, Writing-original draft. Ruijiao Lei: Writing-review & editing. Feiyang Ma: Methodology. Lu Yu, Lanlan Wang: Data processing, Formal analysis. Shangzhi Xu, Yunhua Hu, Linjia Sun: Software, Visualization, Supervision. Wenwen Zhang, Haixia Wang: Validation, Conceptualization. Li Zhang: Resources.

Reference

[1] Li L, Tian H, Zhang B, et al. Prediction for distant metastasis of breast cancer using dynamic contrast-enhanced magnetic resonance imaging images under deep learning(J). Computational Intelligence and Neuroscience, 2022, 2022.

[2] Seeff L C, Nadel M R, Klabunde C N, et al. Patterns and predictors of colorectal cancer test use in the adult US population(J). Cancer: Interdisciplinary International Journal of the American Cancer Society, 2004, 100(10): 2093-2103.

[3] Harbeck N. Breast cancer is a systemic disease optimally treated by a multidisciplinary team(J). Nature Reviews Disease Primers, 2020, 6(1): 30.

[4] Siegel Rebecca L, Miller Kimberly D, Fuchs Hannah E, et al. Cancer statistics, 2021(J). CA Cancer J Clin, 2021, 71(1): 7-33. doi: 10.3322/caac.21654.

[5] Liu W, Zhang R, Ling Y, et al. Automatic recognition of breast invasive ductal carcinoma based on terahertz spectroscopy with wavelet packet transform and machine learning(J). Biomedical optics express, 2020, 11(2): 971-981.

[6] Li J, Zhang B N, Fan J H, et al. A nation-wide multicenter 10-year (1999-2008) retrospective clinical epidemiological study of female breast cancer in China(J). BMC cancer, 2011, 11(1): 1-11.

[7] Ma W, Wang X, Xu G, et al. Distant metastasis prediction via a multi-feature fusion model in breast cancer(J). Aging (Albany NY), 2020, 12(18): 18151.

[8] Dheeba J, Singh N A, Selvi S T. Computer-aided detection of breast cancer on mammograms: A swarm intelligence optimized wavelet neural network approach(J). Journal of biomedical informatics, 2014, 49: 45-52.

[9] Naji M A, El Filali S, Aarika K, et al. Machine learning algorithms for breast cancer prediction and diagnosis(J). Procedia Computer Science, 2021, 191: 487-492.

[10] Battineni G, Chintalapudi N, Amenta F. Performance analysis of different machine learning algorithms in breast cancer predictions(J). EAI Endorsed Transactions on Pervasive Health and Technology, 2020, 6(23).

[11] Li J, Zhou Z, Dong J, et al. Predicting breast cancer 5-year survival using machine learning: A systematic review(J). PloS one, 2021, 16(4): e0250370.

[12] Su H, Liu F, Xie Y, et al. Region segmentation in histopathological breast cancer images using deep convolutional neural network(C)//2015 IEEE 12th International Symposium on Biomedical Imaging (ISBI). IEEE, 2015: 55-58.

[13] Naji M A, El Filali S, Aarika K, et al. Machine learning algorithms for breast cancer prediction and diagnosis(J). Procedia Computer Science, 2021, 191: 487-492.

[14] Support vector machines: theory and applications(M). Springer Science & Business Media, 2005.

[15] Zhe W. The study on prognostic factors of breast cancer based on machine learning method(J). China Digital Med, 2019, 14: 18-20.

[16] Yang Y, Li J, Yang Y. The research of the fast SVM classifier method(C)//2015 12th international computer conference on wavelet active media technology and information processing (ICCWAMTIP). IEEE, 2015: 121-124.

[17] Tran H. A survey of machine learning and data mining techniques used in multimedia system(J). no, 2019, 113: 13-21.

[18] Liaw A, Wiener M. Classification and regression by randomForest(J). R news, 2002, 2(3): 18-22.

[19] Anisha P R, Reddy C K K, Apoorva K, et al. Early Diagnosis of Breast Cancer Prediction using Random Forest Classifier(C)//IOP Conference Series: Materials Science and Engineering. IOP Publishing, 2021, 1116(1): 012187.
[20] Bray F, Ferlay J, Soerjomataram I, et al. Global cancer statistics 2018: GLOBOCAN estimates of incidence and mortality worldwide for 36 cancers in 185 countries(J). CA: a cancer journal for clinicians, 2018, 68(6): 394-424.
[21] Aroef C, Rivan Y, Rustam Z. Comparing random forest and support vector machines for breast cancer classification(J). TELKOMNIKA (Telecommunication Computing Electronics and Control), 2020, 18(2): 815-821.
[22] Cocco D, ElSherif A, Wright M D, et al. Invasive lobular breast cancer: data to support surgical decision making(J). Annals of surgical oncology, 2021, 28: 5723-5729.
[23] Bleyer A, Welch H G. Effect of three decades of screening mammography on breast-cancer incidence(J). New England Journal of Medicine, 2012, 367(21): 1998-2005.
[24] Ahuja A, Al-Zogbi L, Krieger A. Application of noise-reduction techniques to machine learning algorithms for breast cancer tumor identification(J). Computers in Biology and Medicine, 2021, 135: 104576.
[25] Botsikas D, Bagetakos I, Picarra M, et al. What is the diagnostic performance of 18-FDG-PET/MR compared to PET/CT for the N-and M-staging of breast cancer?(J). European radiology, 2019, 29: 1787-1798.
[26] Liu W, Lee M, Wu S N, et al. Using machine learning methods to predict bone metastases in breast infiltrating ductal carcinoma patients(J). Frontiers in Public Health, 2022: 2109.

Click here to access/download
Supporting Information
2.xlsx
