# Supplementary material for: Machine learning-based prediction of distant metastasis risk in invasive ductal carcinoma of the breast
Source: PLoS One. 2025 Feb 26;20(2):e0310410. doi: 10.1371/journal.pone.0310410 (PMC11864521; doi:10.1371/journal.pone.0310410)
Supplement: Attachment — Submitted filename: PONE-D-23-20544R1 Reviewer.pdf [file pone.0310410.s005.pdf]

Reviewer Comments

Journal: PLOS ONE
Manuscript Title: Machine learning-based prediction of distant metastasis risk in invasive ductal carcinoma of the breast
Manuscript ID: PONE-D-23-20544R1
Submission Date: Monday, October 9, 2023

The authors have made a suitable major revision. However, there are some concerns that should be addressed:

Overall Comments:

(1) The conclusions need to be enhanced and the future work should be addressed.
(2) The statistical analysis should be more descriptive.
(3) The following references can be addressed in your study:

a. Balaha, H. M., Antar, E. R., Saafan, M. M., & El-Gendy, E. M. (2023). A comprehensive framework towards segmenting and classifying breast cancer patients using deep learning and Aquila optimizer. Journal of Ambient Intelligence and Humanized Computing, 14(6), 7897-7917.
b. Lee, S. E., Yoon, J. H., Son, N. H., Han, K., & Moon, H. J. (2023). Screening in Patients With Dense Breasts: Comparison of Mammography, Artificial Intelligence, and Supplementary Ultrasound. American Journal of Roentgenology.
c. Baghdadi, N. A., Malki, A., Balaha, H. M., AbdulAzeem, Y., Badawy, M., & Elhosseini, M. (2022). Classification of breast cancer using a manta-ray foraging optimized transfer learning framework. PeerJ Computer Science, 8, e1054.

For the authors in case of the authors got a chance to review the manuscript and submit the revised one after the editor's decision, please, provide a <u>*table*</u> *in the revised manuscript mentioning (1) the comment, (2) the authors' response, and (3) the authors' change (if applicable). Please, consider all of the comments and don't ignore any of them.*

*Please, refer to the attached file "**PONE-D-23-20544R1 Reviewer.pdf**" for the same comments in an organized format.*
