# Supplementary material for: Machine learning-based prediction of distant metastasis risk in invasive ductal carcinoma of the breast
Source: PLoS One. 2025 Feb 26;20(2):e0310410. doi: 10.1371/journal.pone.0310410 (PMC11864521; doi:10.1371/journal.pone.0310410)
Supplement: Attachment — Submitted filename: Response to Reviewers.doc [file pone.0310410.s004.doc]

Dear reviewer:

Thank you very much for your comments and professional advice. These opinions help to improve academic rigor of our article. Based on your suggestion and request, we have made corrected modifications on the revised manuscript. Meanwhile, the manuscript had be reviewed and edited by language services of EDITAGE. We hope that our work can be improved again. Furthermore, we would like to show the details as follows:

**Reviewer 1#**

1. In the context of limited data in this study, data augmentation can be a valuable technique to overcome the scarcity of samples and improve the performance of machine learning models. Data augmentation involves creating additional training examples by applying various transformations to the existing data, while preserving the label information.
   For your study, in the case of breast cancer data, data augmentation techniques can be applied to medical images (such as mammograms or MRI scans) to create new images with subtle modifications. These modifications may include rotation, flipping, zooming, translation, or adding noise, among others. By applying such transformations, the dataset can be expanded, enabling the models to learn from a broader range of scenarios.

**The author’s answer:** Thank you very much for your professional and valuable feedback. We also believe that applying it to images is very meaningful, and we will also consider doing so. However, due to limited research conditions at present. We do not have more relevant data for analysis. In the following research, we will consider expanding the dataset and applying it to a wider range of studies.

2. In addition to addressing the issue of overfitting, it is essential to thoroughly investigate and fine-tune your model's learning rates.

**The author’s answer:** Thank you very much for your professional advice. In our study, we used different indicators including accuracy, precision, recall, AUC value, F1 value, etc. to evaluate the learning effectiveness of the model. The specific research results are presented in 3.4 Model Performance. In addition, we used undersampling to solve the problem of model overfitting, and then used learning curves to evaluate the final fitting effect.

**Reviewer 2#**

1. [KEYWORDS] The keywords (i.e., index terms) should be sorted in alphabetical order.

**The author’s answer:** Thank you for pointing this out. The keywords has been sorted alphabetically and modified.

2. [ABSTRACT] The abstract should contain the best-achieved results from the performed experiments.
3. [ABSTRACT] The abstract should reflect the contributions of the manuscript. I suggest rewriting it.

**The author’s answer:** Thank you for your suggestion. We have rewritten the abstract section, which includes the best results of the experiments conducted and reflects the contributions of the manuscript. The changes to the manuscript are given in the blue text.

4. [INTRODUCTION] The authors should provide a clear problem definition and contributions in the introduction section.

**The author’s answer:** Thank you for your professional suggestion. We have made changes to the introduction and provided a definition of the problem, and we provided the contributions of the manuscript at the end of the introduction. The changes and additions to the introduction are given in the yellow text.

5. [RESEARCH QUESTION] Where is the research question and research gap?
6. [RESEARCH QUESTION] The research question is not well-formulated or is poorly motivated, and the paper does not provide new insights or information that is not already known.

**The author’s answer:** Thank you very much for your professional and valuable feedback. We propose a research gap in the fourth paragraph of the introduction. We have compared (Table 1) with other relevant studies to reflect the current research gap in predicting the risk of IDC cancer metastasis. At the same time, we have proposed our scientific questions to fill this research gap. The changes to the manuscript are given in the yellow text.

7. [RELATED WORK] Where are the related studies? They should be declared in a separate section.
8. [RELATED WORK] A table of comparisons should be added at the end of the related studies section to praise the pros. and cons. of them. The year column should be added and they should be ordered by it.

**The author’s answer:** Thank you very much for your professional and valuable advice. After reading and consulting many literatures, we sorted out the research of machine learning on breast cancer prediction. We present it in the manuscript through Table 1.

9. [EQUATIONS] The authors should follow the journal authors’ guidance in writing the equations, symbols, and variables. Please, refer to the authors guidelines on the journal official website.
10. [EQUATIONS] Where are the equations of the used metrics?

**The author’s answer:** Thanks for your careful checks. Based on your comments, we have made the corrections to make the equations conform to the journal author's guide. The equation for the indicators used is given in the section '2.4 Evaluation indicators'.

11. [DATASETS] Samples from the used dataset should be added and annotated.

**The author’s answer:** Thank you for your comments. We have submitted all datasets in the attachment, including raw data, datasets after inclusion and exclusion criteria, training and testing dataset samples, and undersampling dataset samples.

12. [METHODOLOGY] The suggested approach is not clearly discussed. More scientific details should be added.
13. [METHODOLOGY] What are the used equations in the suggested approach? In other words, how the suggested approach is derived?
14. [METHODOLOGY] Where is the overall pseudocode? Flowchart? of the suggested approach?

**The author’s answer:** We sincerely appreciate the valuable comments. We discussed the proposed method in detail in '2.3 Model Establishment', adding some scientific details and main equations (Equation 2). The overall pseudo code is presented in Table 5.

15. [EXPERIMENTS] The working environment (i.e., software and hardware) should be declared and added to a table.
16. [EXPERIMENTS] The experimental configurations (i.e., settings) should be declared and added to a table.
17. [EXPERIMENTS] What are the criteria for selecting the experimental configurations?
18. [EXPERIMENTS] More experiments should be conducted using different configurations.
19. [EXPERIMENTS] Where is the tabular representation of the reported results?
20. [EXPERIMENTS] Why did not the authors compare their approach with others in a table?
21. [EXPERIMENTS] Why did not the authors compare their approach with another approach to compare the suggested approach efficiency and applicability?
22. [EXPERIMENTS] Where is the detailed and statistical discussion of the reported results?
23. [EXPERIMENTS] More experiments should be conducted using a different dataset to prove the generalization.

**The author’s answer:** We sincerely appreciate the valuable comments. For the 'EXPERIMENTS' question, we provide the following answers:

[15-16] The experimental environment configuration, detailed information on the software package used for machine learning model development, and basic learner parameters information are added to Tables 3, 4, and 6, respectively.

[17] The criteria for selecting experimental configurations are to strive to achieve optimal model performance by setting the above parameters during model training.

[18] We used different parameters to conduct more experiments during the code running process, and ultimately presented the best results in the article.

[19] Thank you very much for your proposal. We have added Tables 7, 8, and 9 to the results section to illustrate the distribution information of the data. Figures 5-8 are the main results of evaluating and comparing the performance of the model.

[20-21] Thank you very much for your professional and valuable advice. After reading and consulting many literatures, we sorted out the research of machine learning on breast cancer prediction. We present it in the manuscript through Table 1.

[22] Thank you for your comments. We conducted a more detailed and in-depth discussion on the results of the report in the discussion section. We updated the background description of breast cancer with reference to the latest literature (273-278 lines); We reorganize and analyze the results (lines 300-322); Discuss in detail the results of univariate and multivariate logistic regression analysis (lines 343-367). The additions to the discussion are given in the red text, and the changes to the discussion are given in the green text.

[23] We appreciate your careful reading of our manuscript and your professional feedback. We tested the model in our study using data from only 20% of the internal test set. There is no external dataset to validate it, which is the main limitation of this study. We strongly agree to use some external data to validate our model. We apologize for the fact that there is currently no available data to conduct the experiments. We will definitely address this limitation in the future if we have the opportunity.

24. The manuscript does not provide a clear contribution to the field of research.

**The author’s answer:** We explained in the last paragraph of the introduction the contribution of the manuscript to the research field. The additions to the discussion are given in the blue text(lines 292-299).

25. The manuscript does not provide a clear justification for the research.

**The author’s answer:** Thank you very much for your question. Our study aims to establish an efficient machine learning model to predict the risk of cancer metastasis in IDC patients. Thereby assisting clinicians in making more rational clinical decisions and enabling patients to receive treatment earlier, which is useful for breast surgeons to quickly and accurately determine whether cancer metastasis has occurred in patients with IDC. We have introduced both the introduction and discussion sections. In addition, we also focused on the performance of single machine learning models and multi algorithm fusion machine learning models, and found that the fusion model has stronger learning performance.

[RESULTS] The authors have not provided adequate visual aids, such as graphs or tables, to help readers understand the data.

**The author’s answer:** Thank you very much for your comments. Based on your suggestion, we have added more tables in the article, such as Tables 7-9. Compared to the initial draft, we have added a total of 9 tables as visual aids, which can help readers understand the data.

26. [ABBREVIATIONS] The authors should add a table of abbreviations in the revised manuscript.
27. [SYMBOLS] The authors should add a table of symbols in the revised manuscript.

**The author’s answer:** Thank you for your suggestion. We have added a list of abbreviations (Table 2).

28. [CONCLUSIONS] The conclusions in this manuscript are primitive. Please, write your conclusions.

**The author’s answer:** Thank you very much for your comments and professional advice. We have made changes to the conclusion section to make it more concise and highlight the key points.

29. [REFERENCES] There are no citations for many sentences in the manuscript. Why? Please check.
30. [REFERENCES] The references should be written in the same style following the journal authors’ guidance.
31. [REFERENCES] Recent citations from 2021 and 2022 should be added to the manuscript.

**The author’s answer:** We are really sorry for our careless mistakes. We have re reviewed the article and corrected this error. Added citation identification to quoted sentences. And modify the reference format according to the requirements of the journal author's guide. Added some latest references from 2021 and 2022. Such as references 1, 2, 13, 18, 23, 30, 33, 35, 36, etc.

32. [PROOFING] The authors should get editing help from someone with full professional proficiency in English.
33. [PROOFING] The manuscript should be checked again to fix any typos such as missing spaces and commas.

**The author’s answer:** Thanks for your careful checks. We are sorry for our carelessness. We first rechecked the manuscript and corrected typos, punctuation errors, and missing spaces. We have invited a professional polishing agency to help polish our manuscript. The editing certification will be provided in the attachment. And we hope the revised manuscript could be acceptable for you.

34. [CONSISTENCY] The manuscript structure is too short. It must be elaborated in their applied technology as should support more rigorous technical aspects.
35. [CONSISTENCY] Some paragraphs are wrapped in more than 10 lines. They should be split concisely.

**The author’s answer:** We greatly appreciate your valuable advice. Based on your suggestion, we have added some article structures. For example, in the results section, we have added '3.1 Demographic characterization' and '3.2 One-way logistic regression analysis' to make the manuscript richer in content and more complete in structure. We have re edited some paragraphs in the manuscript with more than 10 lines to make them concise and concise.

36. [NOVELTY] What is the novelty of the suggested approach?

**The author’s answer:** As each learning model has its own advantages and disadvantages. For example, the XGBoost algorithm adds a regular term to the objective function to control the complexity of the model. It makes the learned model simpler and prevents overfitting. However, this modeling algorithm has too many parameters and is complex to tune. On the contrary, random forest learning model training can be highly parallelized, fast training speed, high efficiency. However, it is prone to overfitting in some noisy classification problems and or regression problems. Then, by constructing a machine learning model that fuses several models together, we can maximize the guarantee that this new model has the maximum advantages and minimum disadvantages. In addition, to the best of our knowledge, it is relatively rare to use this model for predicting IDC transfer risk.

37. [LIMITATIONS] What are the limitations of the current study? It should be added in a separate section.

**The author’s answer:** The main limitation of this study is that the data used are limited by region, volume and subjectivity of feature selection, which constrains the final experimental results. In addition, this is a retrospective study that needs to be validated by prospective studies. We have placed research limitations in a separate section, see '6 Research limitations' in the manuscript.

**Reviewer 3#**

Considering the type of study that is retrospective, the project has no ethical problems. Your project is excellent and I hope that it will increase the life expectancy of cancer patients in the future.

**The author’s answer:** Thank you very much for your endorsement of our project. We are very honored by your praise. We have reviewed this article again and carefully revised and supplemented it, making every effort to make it more scientific and rigorous.

**Reviewer 4#**

1. The paper is well-written and easily understandable.

**The author’s answer:** We feel very honored by your compliments. We have re-examined the article and made careful changes and additions. We'll keep working on it.

2. Authors gave a big part to the Model's definition and presentation, We think they should revisit this part to make it smaller, all the used algorithms are well known.

**The author’s answer:** We think this is an excellent suggestion. We have made changes to the presentation part of the model's definition. In the part of the manuscript where the font is marked blue.

3. A part of the dataset presentation is essential to simplify the problem understanding and make the model's interpretation significant and efficient.

**The author’s answer:** Thanks for your suggestion. As per your suggestion, we have shown the information about the dataset in the section '3.1 Demographic characterization' through Table 6 and Table 7. In addition, the original dataset information, training set and test set data are uploaded through attachments.

4. Are all features important? If Yes why? If No why authors didn't look for a features selection method to optimize the learning process and complexity?

**The author’s answer:** Thank you very much for your question. We explain the issue as follows: Not all characterizing variables are important. In the revised manuscript, we filtered the feature variables by univariate and multivariate logistic regression methods. Statistically significant features were selected for analysis. The specific results can be seen in Table 8.

5. We think that the authors have to explain more about the added value and the importance of using machine learning in this problem and how their solution can help doctors.

**The author’s answer:** We sincerely appreciate your valuable and professional advice. We first introduced the severity of cancer metastasis in IDC in the introduction, so it is important to diagnose whether metastasis occurs as soon as possible. Our work goal is to design and implement a web-based cancer screening tool to help healthcare teams make screening decisions. Users or doctors submit diagnostic forms with input features, and our proposed model will be used to diagnose the cancer metastasis status of the subjects.

6. We recommend that the literature review be in a table containing recent works on the same topics, used models, results, and limitations

**The author’s answer:** Thank you very much for your professional and valuable feedback. We propose a research gap in the fourth paragraph of the introduction. We have compared (Table 1) with other relevant studies to reflect the current research gap in predicting the risk of IDC cancer metastasis.

7. Authors have to present all models configuration, that make the research reproduction possible

**The author’s answer:** We submit the experimental environment configuration (Table 2), detailed information on software packages used for machine learning model development(Table 3), pseudocode for Machine Learning Models(Table 4), base learner parameters(Table 5) in revised manuscript. Hoping to make research reproduction possible.

8. All used features should be clearly presented

**The author’s answer:** Thanks for your comments. We added the clinical and pathological characteristics of the study population in the revised manuscript, and all the characteristic variables used are presented in Tables 6 and 7.

9. We recommend the use of a schema to present the research process

**The author’s answer:** Thanks for your suggestion. We show the data inclusion and exclusion process through Fig 1, the modeling process through Fig 2, the pseudo-code for building the model through Table 4, and the basic learner parameters through Table 5.

**Reviewer 5#**

1. The authors did not provide a list of features used to train their model. They need to show the name, type, and specification of features in a table.

**The author’s answer:** We feel great thanks for your professional review work on our article. In response to your suggestion, we have provided lists showing the feature variables and showing distributional information for each feature. Table 6 shows the distribution of clinical and pathologic features for the overall data study population. Table 7 shows the distribution of features on the training and test sets.

2. The authors mentioned that they used an 8:2 ratio to split the database into train/test sets, but they did not report any results on the model's performance on the 20% unseen test dataset.

**The author’s answer:** Thank you very much for your question. We explain this issue as follows: We explain this problem as follows: we do this by dividing the overall data into a training set, which is used to train the model, and a 20% internal test set, which is used to evaluate the trained model. The section '3.4 Model performance evaluation' in the manuscript shows the results of model performance evaluation on the test set.

3. The authors mentioned in some parts of the manuscript that their original dataset was unbalanced, and they used an undersampling approach to address this issue. However, they did not provide any information on the original size of the dataset or the size of the dataset after downsampling.

**The author’s answer:** We are very sorry for neglecting this part of the content, and thank you very much for your professional reminder. We will actively provide all information about the data in the attachment, including the original dataset, the dataset filtered according to inclusion and exclusion criteria, the undersampling dataset, training set, and test set data.

4. In line 114, the authors mentioned that they used a "small number of features" to train their model. What are the names of these features? They need to be shown in a table.

**The author’s answer:** Thank you very much for your question. We explain this issue as follows: The few features we mentioned here refer to the features that have been screened through univariate and multivariate analysis, and these features have statistical differences from the outcome variables. The use of these filtered features instead of all features is because it can better improve the generalization ability of the classifier and avoid overfitting of the data.

5. The authors claimed that they wanted to predict the "risk" of distant metastases, yet it seems that the models they trained are binary models that have 0/1 output. Usually, for risk prediction, the model needs to have a continuous output to show the probability of an event.

**The author’s answer:** We sincerely appreciate your professional questions and suggestions. Our explanation for this is that the outcome variable of our study is M. In clinical practice, M staging is divided into M0 and M1, which are binary variables, where M0 represents no metastasis and M1 represents metastasis. So, our research also uses machine learning to predict whether patients have distant metastasis of cancer. Based on your suggestion, we are considering changing the 'risk prediction' in the title to 'whether there has been a transfer' to make our research more rigorous.

6. In Figure 4, what do M0 and M1 represent?

**The author’s answer:** Thank you very much for your question. We explain this issue as follows: Clinically, breast cancer usually uses TNM staging. T represents tumor size, N represents whether there is lymph node metastasis, and M represents whether there is distant metastasis. Here, M0 and M1 represent non-transference and transfer, respectively. It means our ending variable.

7. In Figure 5, the details of the features need to be made clear in a table, as mentioned in the comment above.

**The author’s answer:** Thank you very much for your comments. Based on your suggestion, we have added Tables 6 and 7 to present the details of the features.

8. In Figure 6, the confusion matrix needs to be shown in normalized form to make it more readable.

**The author’s answer:** Thank you very much for your professional advice. We have reworked the confusion matrix diagram(Fig 5) and increased the resolution to present it in a normalized form to make it readable.

9. In Figure 8, a table should be used instead of a figure.

**The author’s answer:** Thank you very much for your professional and valuable advice. We started using tables to display the mean and standard deviation of each model's various indicators, but due to the small differences in some indicators, such as the average accuracy of the four models being 0.853, 0.841, 0.862, 0.866, and 0.862, respectively. Therefore, their differences cannot be well presented in the table. Therefore, we choose the box plot (Figure 7) to visually see the differences in indicators and to reflect the standard deviation through the size of the 'box', thus judging the stability of the model.

10. In line 249, the authors mentioned that they ranked the importance of 14 features. How did they choose these 14 features?

**The author’s answer:** Thank you very much for your question. We explain this issue as follows: We modified this section by ranking the importance of all the feature variables (13 features in total excluding the ending variable).

11. In line 327, the authors mentioned that there are a few studies that have done the same work. They should reference these studies in their manuscript and compare their approach with theirs.

**The author’s answer:** Thank you very much for your professional and valuable feedback. We propose a research gap in the fourth paragraph of the introduction. We have compared (Table 1) with other relevant studies to reflect the current research gap in predicting the risk of IDC cancer metastasis.

**Reviewer 6#**

1. This article's main concern is its innovation. In what way does this research innovate? There have been a lot of studies using the mentioned machine learning models, and the idea of voting is not new. What makes the introduced method superior?

**The author’s answer:** Thank you very much for your professional question and comment. We explain the question as follows: many current studies have used a single machine learning model to predict survival outcomes and metastasis risk in cancer. However, each learning model has its own advantages and disadvantages. For example, the XGBoost algorithm adds a regular term to the objective function to control the complexity of the model. It makes the learned model simpler and prevents overfitting. However, this modeling algorithm has too many parameters and is complicated to tune. On the contrary, random forest learning model training can be highly parallelized, fast and efficient. However, it is prone to overfitting in some noisy classification and/or regression problems. We choose a single model that is better in most studies, and by constructing a machine learning model that fuses multiple models together, we can maximize the guarantee that this new model has the maximum advantages and the minimum disadvantages. In addition, we try to select features with fewer and more conventional feature variables for prediction while ensuring high accuracy of the prediction results. Our principle is to use fewer variables to produce more accurate predictions. Finally, to the best of our knowledge, the use of the model to predict IDC transfer risk is relatively rare.

2. As I read the article, my initial thought was to find a reason, based on the topic's attractiveness, why this article might attract readers. Unfortunately, I didn't find the article to be well organized and I was not justified in reading it.

**The author’s answer:** Thank you very much for your comments. We tried our best to improve the manuscript and made some changes to the manuscript. We have reorganized the structure of the manuscript. In the data acquisition section (2.1 Data acquisition), we give the data screening process; in the model establishment section (2.3 Model Establishment), we add information about the configuration of the experimental environment and details about the software packages used for the development of the machine learning models, as well as information about the pseudo-code and the learners' parameters; and in the results section we provide information about the machine learning models developed through the adding 3.1 Demographic characterization and 3.2 One-way logistic regression analysis to show detailed information about the features. We have also made major changes to the discussion section to make it more organized. And we hope the revised manuscript could be acceptable for you.

3. Despite the fact that a lot of research has been conducted in this field, the authors did not compare other methods. As a result, it cannot be justified.

**The author’s answer:** Thank you very much for your professional and valuable feedback. We propose a research gap in the fourth paragraph of the introduction. We have compared (Table 1) with other relevant studies to reflect the current research gap in predicting the risk of IDC cancer metastasis.

4. The abstract of the article has not been prepared properly. The abstract should begin with a short introduction containing the assumption and challenge of the problem, followed by the proposed method to solve the problem, the results (quantitatively and qualitatively), and the conclusion.

**The author’s answer:** Thank you for your suggestion. We have rewritten the abstract section, which includes the best results of the experiments conducted and reflects the contributions of the manuscript.

5. There is a lack of clarity and depth in the introduction related work. The authors should organize them professionally. Furthermore, the references are outdated.

**The author’s answer:** Thank you very much for your professional and valuable comments. We have made changes to the introductory section. The introductory section includes the current status of breast cancer and prevalence, leading to the occurrence of cancer metastasis as a big problem facing breast cancer. It then presents IDC, a pathological type of breast cancer with a high metastatic rate, as the target disease to be studied. In the third paragraph we describe the current state of machine learning and machine learning combined with healthcare problems, followed by proposing our solution. In the last paragraph we give the contribution made by the manuscript. In addition, we added some latest references from 2021 and 2022. Such as references 1, 2, 13, 18, 23, 30, 33, 35, 36, etc.

6. Does the authors' assertion that they implemented the method on this dataset justify the method's generalizability?

**The author’s answer:** We appreciate your careful reading of our manuscript and your professional feedback. We tested the model in our study using data from only 20% of the internal test set. There is no external dataset to validate it, which is the main limitation of this study. We strongly agree to use some external data to validate our model. We apologize for the fact that there is currently no available data to conduct the experiments. We will definitely address this limitation in the future if we have the opportunity.

7. Results are poorly presented. Can the classification process be affected by changing parameters? Both qualitative and quantitative results are desired by the reader.

**The author’s answer:** Thank you very much for your comments. According to this problem, we have made the following changes: firstly, we have added tables in order to present the results more clearly, as in Table 6-8. secondly, we have increased the resolution of the images. Changing the parameters will have some effect on the results, so the basic learner parameters (Table 5) that we used during the experiments are the ones that can optimize the performance of each model.

8. The article should be carefully revised by the authors and possible typos and writing errors should be corrected.

**The author’s answer:** Thanks for your careful checks. We are sorry for our carelessness. We first rechecked the manuscript and corrected typos, punctuation errors, and missing spaces. We have invited a professional polishing agency to help polish our manuscript. The editing certification will be provided in the attachment. And we hope the revised manuscript could be acceptable for you.

9. In order to understand the disadvantages of the proposed method, it is recommended to include some figures showing that the proposed method does not produce the expected results. It is also better to mention the disadvantages of the proposed method.

**The author’s answer:** We very sincerely appreciate your professional and valuable comments. Our proposed method also has some drawbacks, such as the fact that the results of the learning curve are not the best, which is also mentioned in the manuscript (line 243-246 and Fig. 8).

10. The figures (e.g., Figures 8 and 10) are of poor quality. The authors should include more high-quality figures in the text. It is possible to save images in EMF format to ensure better quality.

**The author’s answer:** We are really sorry for our careless mistakes. Thank you for your reminder. We have reviewed and modified all the images in the article to achieve a resolution of 300dpi per image. All images are uploaded through the attachment (Figure).

11. There will be a better version of this work with better preparation, such as comparative results, different method output figures, etc.

**The author’s answer:** Thank you for your comments. We have made significant modifications to the presentation of the results. Use tables and numbers to re present the results. Make the results more readable.

12. The article appears to have been written in a hurry, has a low level of innovation, and unfortunately, the professor supervisor did not check the article thoroughly. Although this research has some basic flaws, I believe it still faces some basic challenges.

**The author’s answer:** Thank you very much for your feedback. We have re reviewed the article and made significant changes based on your suggestion. We have rewritten the abstract first; Make more organized changes to the introduction and provide contributions to the manuscript; Provided more detailed and complete data information; Provided information such as working environment and experimental configuration; In the results section, we added the display of features (Tables 7-9); In the discussion section, we provided a more detailed analysis.And we hope the revised manuscript could be acceptable for you.
