# Supplementary material for: Machine learning-based prediction of distant metastasis risk in invasive ductal carcinoma of the breast
Source: PLoS One. 2025 Feb 26;20(2):e0310410. doi: 10.1371/journal.pone.0310410 (PMC11864521; doi:10.1371/journal.pone.0310410)
Supplement: Attachment — Submitted filename: Response to Reviewers.doc [file pone.0310410.s006.doc]

Dear reviewer:

Thank you very much for your comments and professional advice. These opinions help to improve academic rigor of our article. Based on your suggestion and request, we have made corrected modifications on the revised manuscript. We hope that our work can be improved again. Furthermore, we would like to show the details as follows:

**Reviewer #2**

1. The conclusions need to be enhanced and the future work should be addressed.

**The author’s answer:** Thank you very much for your professional and valuable feedback. We have rewritten the conclusion section to make it more comprehensive. The changes to the manuscript are given in the blue text. As for the next steps, our primary focus will be on external validation, a task we are actively pursuing.

2. The statistical analysis should be more descriptive.

**The author’s answer:** Thank you very much for the positive and constructive comments regarding our paper. We provided a detailed description of the methodology section, covering steps such as data acquisition, data inclusion, exclusion, and preprocessing, as well as model establishment. To ensure readers have a clear understanding of our statistical analysis workflow, we utilized figures for a more concise presentation of results, such as Figures 1, 2, and 3.

3. The following references can be addressed in your study:a.Balaha, H. M., Antar, E. R., Saafan, M. M., & El-Gendy, E. M. (2023). A comprehensive framework towards segmenting and classifying breast cancer patients using deep learning and Aquila optimizer. Journal of Ambient Intelligence and Humanized Computing, 14(6), 7897-7917.b.Lee, S. E., Yoon, J. H., Son, N. H., Han, K., & Moon, H. J. (2023). Screening in Patients With Dense Breasts: Comparison of Mammography, Artificial Intelligence, and Supplementary Ultrasound. American Journal of Roentgenology.c.Baghdadi, N. A., Malki, A., Balaha, H. M., AbdulAzeem, Y., Badawy, M., & Elhosseini, M. (2022). Classification of breast cancer using a manta-ray foraging optimized transfer learning framework. PeerJ Computer Science, 8, e1054.

**The author’s answer:** We sincerely appreciate the valuable comments. As suggested by the reviewer, we consider the references you recommended to be valuable for our research. Therefore, we have cited them in the paper (references 2, 5, and 49, respectively).

**Reviewer #3**

According to the corrections that were suggested by different reviewers , this article seems to be suitable for publishing.

**The author’s answer:** We would like to thank you for your professional review work, and for the positive feedback.

**Reviewer #5**

Thanks. I didn't see the "data availability" exploitation in the manuscript. That would be good to add a statement in the manuscript to show how readers can access to the data.

**The author’s answer:** We thank the reviewer for bringing this to our attention. We have appended a "data availability" statement at the end of the conclusion section and uploaded the data to Figshare. The original data can be directly accessed through the link we provided.

**Reviewer #6**

There have been many solutions to the previous problems. The authors should take care that the responsibility for the results and validation of the results lies with them alone. Figures and references should be carefully reviewed. The references to the equations, as well as the definitions of some of their parameters, should also be carefully checked.

**The author’s answer:** We thank the reviewer for the helpful comments and appreciation of our work. We meticulously reviewed the figures, reference materials, as well as the references for equations and definitions of certain parameters, making appropriate modifications. Thanks to the reviewer for this careful comments.
